# Leukaemia cell of origin identified by chromatin landscape of bulk tumour cells

**DOI:** 10.1038/ncomms12166

**Published:** 2016-07-11

**Authors:** Joshy George, Asli Uyar, Kira Young, Lauren Kuffler, Kaiden Waldron-Francis, Eladio Marquez, Duygu Ucar, Jennifer J. Trowbridge

**Affiliations:** 1 The Jackson Laboratory for Genomic Medicine, 10 Discovery Drive, Farmington, Connecticut 06032, USA; 2 The Jackson Laboratory for Mammalian Genetics, 600 Main Street, Bar Harbor, Maine 04609, USA

## Abstract

The precise identity of a tumour's cell of origin can influence disease prognosis and outcome. Methods to reliably define tumour cell of origin from primary, bulk tumour cell samples has been a challenge. Here we use a well-defined model of *MLL*-rearranged acute myeloid leukaemia (AML) to demonstrate that transforming haematopoietic stem cells (HSCs) and multipotent progenitors results in more aggressive AML than transforming committed progenitor cells. Transcriptome profiling reveals a gene expression signature broadly distinguishing stem cell-derived versus progenitor cell-derived AML, including genes involved in immune escape, extravasation and small GTPase signal transduction. However, whole-genome profiling of open chromatin reveals precise and robust biomarkers reflecting each cell of origin tested, from bulk AML tumour cell sampling. We find that bulk AML tumour cells exhibit distinct open chromatin loci that reflect the transformed cell of origin and suggest that open chromatin patterns may be leveraged as prognostic signatures in human AML.

## Abstract (web summary)

A tumour's cell of origin may influence tumour progression and response to therapy. Here, the authors demonstrate that the cell of origin determines the aggressiveness of AML in a mouse model and identify unique biomarkers of the specific leukaemia cell of origin by profiling open chromatin regions of AML samples.

## Graphical abstract

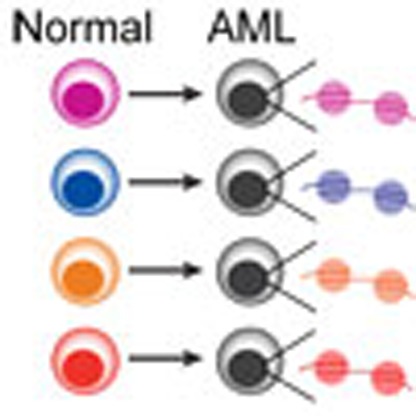

Accumulating evidence suggests that the cellular context or ‘cell of origin' in which a genetic lesion occurs contributes to the emergence of distinct tumour subtypes^1^. Identifying cell of origin may allow better prediction of tumour behaviour, including tumour progression and response to therapeutics. Current methods to retrospectively identify cell of origin from tumour cell samples are limited. Histology, cytogenetics and cell surface marker staining may be insufficient to accurately predict cell of origin when the initiating cells are phenotypically similar and/or exist within the same tissue. Transcriptional profiling has been used to substantiate the tumour cell of origin model; however, technical variation due to platform differences and batch effects between studies have a significant impact on the ability to consistently apply expression signatures to clinical samples^2^. Recently, accurate determination of tumour cell of origin was achieved based on the distribution of mutations along the genome of tumour cells^3^. This approach is most accurate when putative cells of origin have distinct gene expression profiles, chromatin organization and lineage fate. For tumours arising from cells along a differentiation spectrum within the same tissue, most abundantly characterized in haematological malignancies^4^,^5^,^6^, it remains a challenge to determine cell of origin from bulk circulating tumour cells.

Epigenetic modification is one conduit through which developmental origins of a cell are retained during differentiation, and potentially, transformation. On the basis of striking cell type specificity of chromatin structure^7^,^8^, we hypothesized that assessment of open chromatin in bulk tumour cells with matched cells of origin would reveal novel epigenetic biomarkers of tumour cell of origin. To evaluate this hypothesis, we used a well-established and clinically relevant model of acute myeloid leukaemia (AML) driven by expression of the fusion oncogene *MLL-AF9 (MA9)*^9^,^10^. Chromosomal translocations of the *MLL* (mixed lineage leukaemia) gene on human chromosome 11q23 are found in ∼5% of adult and 50% of paediatric AML cases^11^,^12^. The *MLL* gene encodes a methyltransferase, which modifies histones to control the expression of target genes including the *HOX* gene family^13^. AML with t(9;11)(p22;q23) translocation giving rise to *MA9* is the most common MLL-rearranged AML. Among AML cases with t(9;11) there is great clinical heterogeneity. Studies in mice have demonstrated that MA9 can confer self-renewal activity to committed myeloid progenitors as well as transform HSCs^4^, supporting use of this model to test cell of origin in AML development. Here we test the impact of cell of origin on AML development starting from cells within a differentiation spectrum from stem cells through lineage-committed progenitor cell types. We compare both global transcriptome and epigenome (open chromatin) signatures of the resulting leukaemias to their respective cell of origin, to evaluate global changes in chromatin structure that occur during the process of transformation, and how these changes differ when AML is initiated from distinct cell types.

## Results

### Transformed cell of origin dictates growth of AML cells

**Figure 1:**
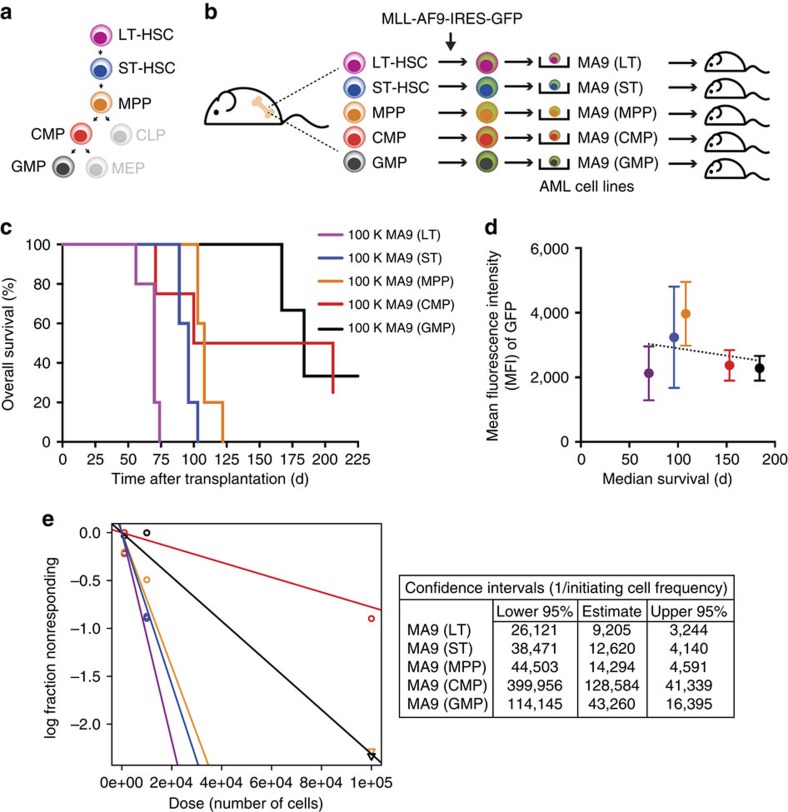
Cell of origin determines potency of *in vitro-*derived MA9-driven AML. (**a**) Schematic diagram of the primitive haematopoietic hierarchy. Colour code: purple for long-term HSCs (LT-HSC), blue for short-term (ST)-HSC, orange for multipotent progenitors (MPP), red for common myeloid progenitors (CMP), black for granulocyte macrophage progenitors (GMP). (**b**) Schematic diagram of experimental design to test *in vitro* transformation of distinct cells of origin by MA9. (**c**) Overall survival of mice transplanted with 100 K MA9-transformed cells from distinct cells of origin (*n*=5 per group). Technical replicates of 5 mice were each transplanted with 100 K cells from one set of cell lines. Trend and significance were replicated once using a set of independently derived cell lines. Log-rank test; *P*<0.0001. (**d**) Correlation between mean fluorescence intensity (MFI) of GFP in leukaemia cell lines and median survival (days) of mice transplanted with these lines (*n*=5 per group). Centre values indicate mean. Error bars indicate s.e.m. Pearson's *r*=−0.2653, not significant. (**e**) Limiting dilution analysis of leukaemia cell lines from distinct cells of origin (*n*=5 per group, per cell dose). Technical replicates of five mice were each transplanted with 100 K, 10 K or 1 K cells from one set of cell lines. Trend and significance were replicated once using a set of independently derived cell lines. Confidence intervals (1 per initiating cell frequency) were calculated with ELDA software^50^. Pearson's *χ*^2^-test; *P*=0.00145.

To test the impact of cell of origin on leukemogenesis, we isolated enriched populations of haematopoietic stem and progenitor cells, including long-term HSCs (LT-HSCs), short-term HSCs (ST-HSCs), multipotent progenitors (MPPs), common myeloid progenitors (CMPs) and granulocyte macrophage progenitors (GMPs) (Fig. 1a, Supplementary Fig. 1a,b). Transformed cell lines were derived from independent biological replicates (*n*=2) following retroviral transduction with MLL-AF9 (MA9) and GFP reporter (Fig. 1b), and transplanted into sublethally irradiated recipient mice (*n*=5 recipients per cell line). We observed distinct *in vivo* penetrance and rate of AML development in these mice (Fig. 1c). MA9 cell lines derived from LT-HSCs (MA9 (LT)) were the most aggressive, with complete penetrance and a median survival of 70 days (70d) post transplant. In pair-wise comparisons, this was significantly different from overall survival of MA9 (ST) (median 96d, log-rank test *P*=0.002), MA9 (MPP) (median 108d, log-rank test; *P*=0.002), MA9 (CMP) (median 153d, log-rank test; *P*=0.0190), and MA9 (GMP) (median 184d, log-rank test; *P*=0.0116; Supplementary Table 1). To evaluate whether these distinct growth properties were associated with different levels of *MA9* expression, we evaluated mean fluorescence intensity of GFP, which is correlated to the level of *MA9* expression (Supplementary Fig. 1c). GFP intensity did not correlate to median survival time (Fig. 1d), suggesting that differing levels of *MA9* expression do not account for differences in tumour aggressiveness. Altogether, these data suggest that cell of origin impacts the rate of AML development. Specifically, HSC-derived AMLs were the most aggressive and differentiated progenitor cell-derived AMLs were the least aggressive.

To quantitate the frequency of leukaemia-initiating cells in MA9 cell lines derived from distinct cells of origin, we performed limiting dilution transplantation. Using Poisson statistics, the quantitation estimate of leukaemia-initiating cell frequency was significantly higher in MA9 (LT) versus MA9 (CMP) or MA9 (GMP) (Pearson's *χ*^2^-test; *P*=0.000643 and *P*=0.0501, respectively; Fig. 1e, Supplementary Tables 2,3). These results suggest that the cell of origin impacts leukaemia-initiating cell frequency, with a spectrum from HSC-derived AMLs having the highest leukaemia-initiating cell content to the most differentiated progenitor cell-derived AMLs having the lowest leukaemia-initiating cell content, consistent with our observations of the distinct penetrance and rate of AML development based on cell of origin.

### *In vivo* AML development is dependent on cell of origin

**Figure 2:**
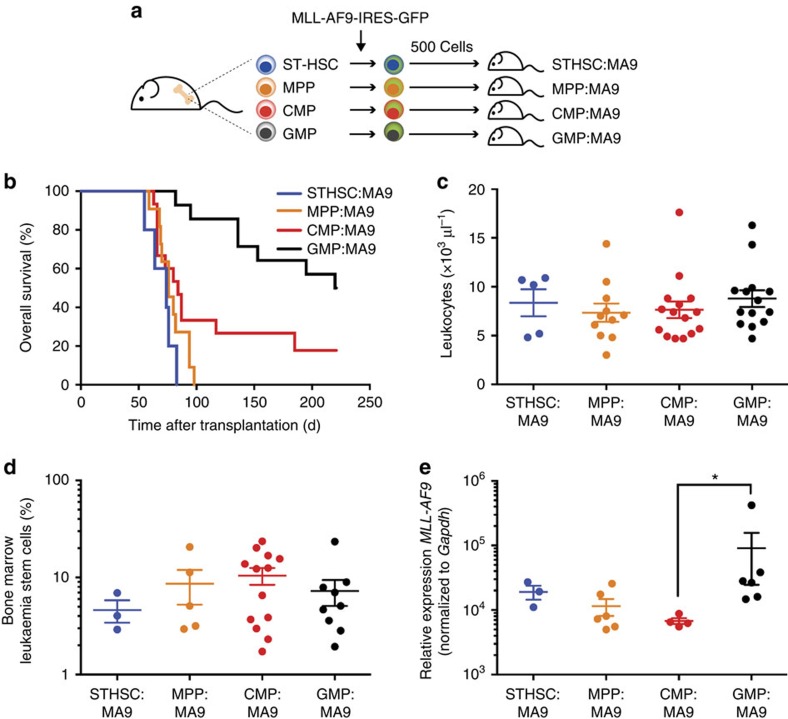
Cell of origin alters tumour aggressiveness of *in vivo*-derived AML. (**a**) Schematic diagram of experimental design to test *in vivo* transformation of distinct cells of origin by MA9. (**b**) Overall survival of mice transplanted with 500 *MA9*-transduced cells from distinct cells of origin (STHSC:MA9; *n*=5, MPP:MA9; *n*=11, CMP:MA9; *n*=15, GMP:MA9; *n*=14). Data were collected over three biological replicate experiments. Log-rank test; *P*=0.0004. (**c**) Peripheral blood leukocyte count in terminal mice transplanted with *MA9*-transduced cells (STHSC:MA9; *n*=5, MPP:MA9; *n*=11, CMP:MA9; *n*=15, GMP:MA9; *n*=14). Data were collected over three biological replicate experiments. Centre bars indicate mean. Error bars indicate s.e.m. Kruskal-Wallis test; *P*=0.5622. (**d**) Average frequency of LSCs (L-GMP^20^) in the bone marrow of terminal mice (STHSC:MA9; *n*=3, MPP:MA9; *n*=6, CMP:MA9; *n*=13, GMP:MA9; *n*=9). Data were collected over three biological replicate experiments. Centre bars indicate mean. Error bars indicate s.e.m. Kruskal–Wallis test; *P*=0.7031. (**e**) Relative expression of *MA9* assessed by real-time PCR in splenic leukaemia cells in terminal mice (STHSC:MA9; *n*=3, MPP:MA9; *n*=6, CMP:MA9; *n*=4, GMP:MA9; *n*=6). Data were collected over three biological replicate experiments. Centre bars indicate mean. Error bars indicate s.e.m. Kruskal–Wallis test; *P*=0.0055. Dunn's multiple comparisons test; *P<0.05.

To evaluate the impact of cell of origin on *in vivo* leukemogenesis, haematopoietic stem and progenitor cells were transduced with *MA9* and transplanted immediately into sublethally irradiated recipients (Fig. 2a). To distinguish from cell line-derived leukaemias, we have termed these STHSC:MA9, MPP:MA9, CMP:MA9 and GMP:MA9. We observed distinct *in vivo* penetrance and rate of AML development based on the cell of origin (Fig. 2b). STHSC:MA9 and MPP:MA9 were fully penetrant with a median survival time of 74d and 76d post transplant, respectively. CMP:MA9 and GMP:MA9 were partially penetrant (80 and 50%, respectively), with a median survival time of 84d and 239d. In pair-wise comparisons, overall survival of STHSC:MA9, MPP:MA9 and CMP:MA9 were significantly different from overall survival of GMP:MA9 (log-rank test; *P*<0.0001, *P*<0.0001, *P*=0.0086, respectively), and STHSC:MA9 were significantly different from CMP:MA9 (log-rank test; *P*=0.0358; Supplementary Table 4). Thus, the cell of origin impacts *in vivo* transformation rate and progression of disease, with STHSC:MA9 and MPP:MA9 progressing rapidly, and CMP:MA9 and GMP:MA9 progressing at a slower rate. We did not observe a significant difference in leukaemia-bearing mice with respect to leukocyte count (Fig. 2c), frequency of GFP^+^ cells in the bone marrow (Supplementary Fig. 1d), spleen weight (Supplementary Fig. 1e), or frequency of leukaemia stem cells (LSCs) as defined by the cell surface L-GMP phenotype^9^ (Fig. 2d). Of note, the cell surface L-GMP phenotype has previously been shown by secondary transplantation to enrich for LSC activity using the *MA9* retroviral transduction model starting from both HSC and GMP cells of origin^4^. We did observe distinct expression of the *MA9* transcript in bulk leukaemia cells (Fig. 2e) and LSCs (Supplementary Fig. 1f) based on cell of origin. GMP:MA9 bulk leukaemia cells and LSCs had significantly higher *MA9* expression than CMP:MA9 bulk leukaemia cells and LSCs, respectively, consistent with the observation that higher levels of *MA9* are required to transform GMP cells^14^. Altogether, these data suggest that the gross phenotype of MA9-driven AML from distinct cells of origin is highly similar, consistent with previous reports^4^.

### HSC-derived AMLs have distinct gene expression signature

**Figure 3:**
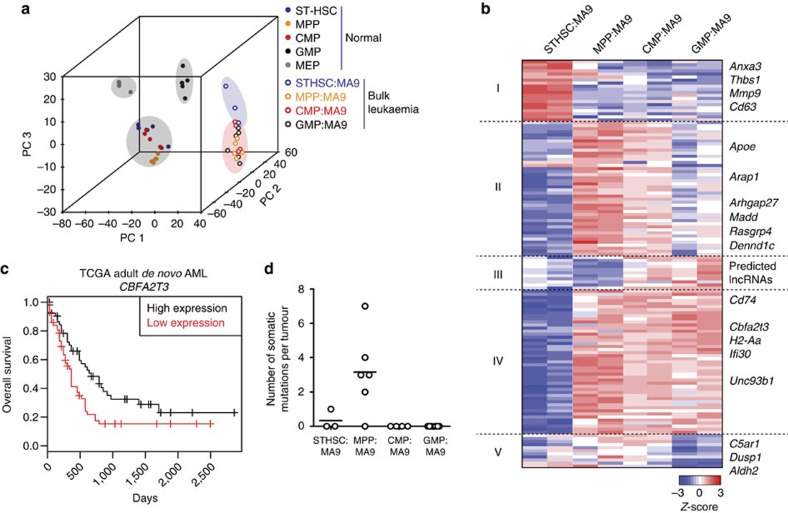
ST-HSC-derived AML has a unique and prognostically relevant gene expression signature. (**a**) Principal component analysis of normal and MA9-transformed haematopoietic stem and progenitor cells (STHSC; *n*=5, MPP; *n*=6, CMP; *n*=5, GMP; *n=*5, MEP; *n*=4, STHSC:MA9; *n*=3, MPP:MA9; *n*=6, CMP:MA9; *n*=4, GMP:MA9; *n*=6). Each sample was obtained from an individual mouse. Data were collected from two biological replicate experiments. Clustering segregates STHSC:MA9 leukemias (blue cloud) from other leukaemias (red cloud). (**b**) K-medioids clustering (*K*=5) of 133 differentially expressed genes in leukaemias based on cell of origin (foldchange >2, FDR<0.05) (STHSC:MA9; *n*=3, MPP:MA9; *n*=6, CMP:MA9; *n*=4, GMP:MA9; *n*=6). Representative biological replicates are shown. (**c**) Overall survival of human AML patients based on expression of *CBFA2T3* (*n*=200). Log-rank test; *P*=0.0113. (**d**) Number of somatic nucleotide variants (SNVs) per tumour derived from distinct cells of origin (STHSC:MA9; *n*=3, MPP:MA9; *n*=6, CMP:MA9; *n*=4, GMP:MA9; *n*=6). Each sample was obtained from an individual mouse. Data were collected from two biological replicate experiments. Kruskal–Wallis test; *P*=0.0019.

To interrogate global gene expression signatures, we performed RNA sequencing (RNA-seq) on *in vivo*-derived bulk leukaemias, as well as non-transformed haematopoietic stem and progenitor cells including LT-HSC, ST-HSC, MPP, CMP, GMP and MEP (megakaryocyte–erythrocyte progenitors). Principal component analysis (PCA) determined that the top three PCs together accounted for ∼80% of the biological variation between samples (Supplementary Fig. 2A). PCA plots revealed that STHSC:MA9 exhibited a unique molecular signature compared with MPP:MA9, CMP:MA9 and GMP:MA9 (Fig. 3a, Supplementary Fig. 2b). *Evi1*, a previously reported proto-oncogene predicting poor prognosis in human AML^4^,^15^,^16^, was significantly higher in MPP:MA9 compared with CMP:MA9 and GMP:MA9 (Supplementary Fig. 2c).

Focusing on differences between leukaemias based on cell of origin, we identified 133 genes that were differentially expressed between at least two leukaemia subtypes with foldchange >2 and false discovery rate (FDR)<0.05 (Fig. 3b, Supplementary Data 1). Differentially expressed genes clustered into five groups based on their expression patterns across samples. GO analysis of Cluster I (up in STHSC:MA9) revealed greatest enrichment of ‘regulation of epithelial cell migration' (*P*=7.19e^−6^, FDR=9.66e^−2^), angiogenesis and vasculature development terms, suggesting STHSC:MA9 may have a greater ability to establish extramedullary disease^17^. Clusters II and IV (down in STHSC:MA9) were enriched for ‘regulation of small GTPase mediated signal transduction' (*P*=5.93e^−5^, FDR=1.99e^−1^) and ‘antigen processing and presentation of peptide antigen via MHC class II' (*P*=2.90e^−8^, FDR=1.95e^−4^), respectively. These data suggest MPP:MA9, with increased expression of Cluster II, have greater activation of small GTPase signalling and may be sensitive to targeting of these pathways^18^. STHSC:MA9, with the lower expression of Cluster IV, may have greater ability to escape from immune recognition^19^, contributing to their aggressive phenotype. Cluster III (up in CMP:MA9 and GMP:MA9) mainly included predicted *lncRNA* genes, and Cluster V (up in MPP:MA9 and CMP:MA9) included genes associated with negative regulation of cell death. To evaluate whether these gene signatures were retained from normal cell of origin or were a consequence of transformation, we compared the expression of these genes in normal ST-HSC, MPP, CMP and GMP (Supplementary Fig. 2d). Cluster I was not highly expressed in normal ST-HSC, but was highly expressed in normal GMP, consistent with the previously characterized gene expression signature common between normal GMP and LSCs^20^. Clusters II and IV were lowly expressed in normal ST-HSC versus normal MPP, CMP and GMP. Clusters III and V were lowly expressed across all normal cell types. Together, these data demonstrate that transcriptional differences in tumours derived from distinct cells of origin can be either transformation-dependent (Clusters I, III and V) or reflect the maturation stage of the cell of origin (Clusters II, IV). Overall, the greatest differences in expression between leukaemias based on cell of origin were found between STHSC:MA9 and MPP:MA9. These data identify a transcriptional signature that may be used to distinguish ST-HSC-derived from progenitor-derived leukaemias.

To evaluate clinical relevance of this signature, we converted each gene to human gene ID and computed prognostic significance of expression level in 200 *de novo* AML patients spanning the major morphologic and cytogenetic subtypes of AML^21^. We observed a statistically significant enrichment of prognostic genes (19% in signature versus 10% in overall, two-sample test for equality of proportions with continuity correction; *P*=0.00018). For example, *Cbfa2t3* was identified as downregulated in STHSC:MA9 versus progenitor-derived tumours (Fig. 3b), and in human AML, low expression of *CBFA2T3* was associated with poor prognosis (Fig. 3c). Kaplan–Meier survival curves and relationship of prognostic genes to cytogenetic risk category are shown in Supplementary Fig. 3.

Our transcriptional profiling data revealed a limitation in robustly distinguishing and segregating progenitor-derived leukaemias. At the foldchange >2 and FDR<0.05 threshold, no differentially expressed genes were observed between MPP:MA9 and CMP:MA9, and only one gene between CMP:MA9 and GMP:MA9 (Supplementary Data 1). On the basis of our *in vitro* and *in vivo* functional assays, there are significantly different potency and penetrance of leukaemias derived from MPP, CMP and GMP, and thus a reliable strategy to identify distinct biomarkers of these leukaemias would be predicted to have impactful prognostic utility.

### Recurrent mutations not associated with cell of origin

A potential driver of differential AML aggressiveness based on cell of origin, and biomarker of cell of origin, is the acquisition of distinct cooperating mutations. Using RNA-seq data, single-nucleotide variants and small insertions and deletions were identified using FreeBayes, a Bayesian genetic variant detector^22^. Variants detected using this method were filtered based on read depth and mapping quality to reduce false positive rate^23^. Germline single-nucleotide polymorphisms (SNPs) identified in C57BL/6 mice using the Sanger Institute SNP resource^24^ and SNPs identified in any of our matched normal cell RNA-seq data were removed. With respect to somatic mutational burden, only MPP:MA9 were found to consistently contain coding mutations (average three mutations per tumour), and these were not found to be recurrent across multiple biological replicates (Fig. 3d, Supplementary Table 5). These findings are consistent with data from The Cancer Genome Atlas (TCGA) showing that *MLL* fusion-driven AML requires fewer cooperating mutations (on average two mutations per tumour) than other AML-initiating events (on average five mutations per tumour)^21^. Overall, these data suggest that there are no recurrent patterns of MA9-cooperating mutations from distinct cells of origin that would account for the observed differential tumour aggressiveness in our model.

### Global chromatin remodelling during MA9 transformation

**Figure 4:**
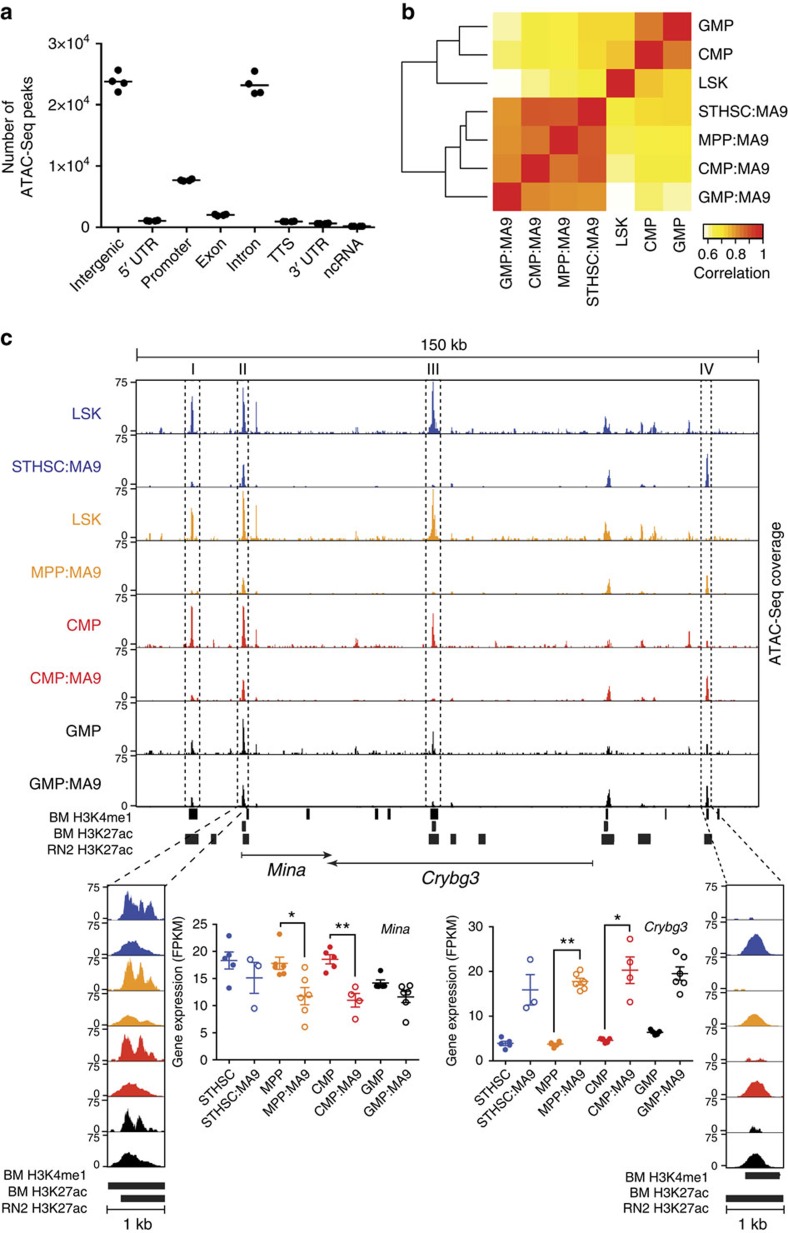
Global chromatin remodelling during MA9 transformation. (**a**) Number of ATAC-seq peaks distributed across annotated features of the genome (*n=*4 biological replicates from individual mice). Centre bars indicate mean. (**b**) Heatmap of Pearson correlation analysis of open chromatin regions in *in vivo*-derived primary bulk leukaemias from distinct cells of origin and their normal cellular counterparts (LSK; *n=*3, CMP; *n*=3, GMP; *n*=2, STHSC:MA9; *n=*2, MPP:MA9; *n*=2, CMP:MA9; *n*=2, GMP:MA9; *n*=2). LSK; Lin^−^ Sca-1^+^ c-Kit^+^, includes ST-HSC and MPP cells. Each sample was obtained from an individual mouse. Data were collected from two biological replicate experiments. (**c**) Normalized ATAC-seq profiles of *in vivo*-derived primary bulk leukaemias from distinct cells of origin and their normal cellular counterparts, showing gain and loss of enhancer elements (dotted lines) around *Mina* and *Crybg3* in a 150-kb region. Single samples are shown. Trends were replicated once with independent biological replicates. Shown at bottom are H3K4me1 and H3K27ac ChIP-seq peak regions in normal murine bone marrow (BM) and H3K27ac peak regions in a MA9/Nras^G12D^ murine AML cell line (RN2). RNA-seq FPKM of *Mina* and *Crybg3* are shown (STHSC; *n*=5, MPP; *n*=6, CMP; *n*=5, GMP; *n*=5, STHSC:MA9; *n*=3, MPP:MA9; *n*=6, CMP:MA9; *n*=4, GMP:MA9; *n*=6). Centre bars indicate mean. Error bars indicate s.e.m. Kruskal–Wallis test; *P*=0.0012 and *P<*0.0001, respectively. Dunn's multiple comparisons test; *P<0.05, **P<0.01.

Recent studies have demonstrated that chromatin landscape reflects both cellular developmental origin as well as its future potential^25^,^26^. To profile chromatin landscape, we performed the assay for transposase-accessible chromatin (ATAC-seq)^27^ on *in vivo*-derived bulk leukaemias from each cell of origin, and compared this with published ATAC-seq data on normal cell types including LSK (Lin^−^ Sca-1^+^ c-Kit^+^, includes ST-HSC and MPP), CMP and GMP by Lara-Astiaso *et al.*^25^ We found that ATAC-seq peaks are most abundant in intergenic and intronic regions, followed by promoter regions (Fig. 4a), suggesting that ATAC-seq offers distinct regulatory information than what can be gained from transcriptional signatures alone. Pearson's correlation values between ATAC-seq samples revealed an overall open chromatin landscape similarity between leukaemia cells regardless of their cell of origin, when compared with their normal cellular counterparts, supporting global changes in chromatin remodelling during MA9 transformation (Fig. 4b). Within the leukaemias derived from distinct cells of origin, STHSC:MA9 and MPP:MA9 were most similar (*r*=0.914), followed by CMP:MA9 (*r*=0.906), with GMP:MA9 being the most distinct (*r*=0.862), consistent with both the hierarchical relationship between the cells of origin and the rates of *in vivo* leukaemia development. To determine whether open chromatin landscape would be more distinct in LSCs derived from each cell of origin, we performed ATAC-seq analysis on purified cells with the LSC phenotype (L-GMP). Pearson's correlation analysis revealed an overall open chromatin landscape similarity between LSCs regardless of their cell of origin (Supplementary Fig. 4). Furthermore, STHSC:MA9 and MPP:MA9 LSCs were most similar (*r*=0.946), followed by CMP:MA9 LSCs (*r*=0.919), with GMP:MA9 LSCs being the most distinct (*r*=0.884), a pattern recapitulating the relationship observed at the bulk tumour level. As larger differences were observed based on cell of origin at the bulk tumour level, and bulk tumour sampling has greater translational potential, we focused further characterization on differences in open chromatin regions at the bulk tumour level.

Significant chromatin remodelling was found to occur during the process of transformation, independent of cell of origin, demonstrated by a comparison of open chromatin regions between bulk leukaemia cells with normal LSK, CMP and GMP (Supplementary Fig. 5a). Overlapping peaks identified between normal and bulk leukaemia cells were found to have a similar range of peak scores compared with unique peaks (Supplementary Fig. 5b), supporting that this relationship is not due to differential sequencing depth. A number of open chromatin regions were specifically retained from cell of origin; present in STHSC:MA9 and LSK only (2,745), MPP:MA9 and LSK only (3,221), CMP:MA9 and CMP only (4,566) and GMP:MA9 and GMP only (904) (Supplementary Fig. 5A). Interestingly, we observed that GMP:MA9 gained 13,592 peaks corresponding to less-differentiated cells of origin (LSK and CMP), while the CMP:MA9 tumours gained 3,210 peaks corresponding to normal LSK. With respect to peaks gained corresponding to more highly differentiated cells of origin, STHSC:MA9 gained 8,339 peaks, MPP:MA9 gained 8,753 peaks and CMP:MA9 gained 787 peaks. These results suggest that global chromatin alterations occur during MA9-induced transformation, including gain, loss and retention of open chromatin regions.

Distinct open chromatin loci were identified between normal and tumour cells. Both loss of open chromatin upstream of the transcriptional start site (Region I), within an intron (Region II) and downstream of the coding sequence (Region III) of the gene *Mina* on chromosome 16, and gain of open chromatin upstream of the neighbouring gene *Crybg3* (Region IV) were observed (Fig. 4c). Examination of chromatin immunoprecipitation sequencing (ChIP-seq) data for the enhancer-relevant histone modifications H3K4me1 (marking poised and active enhancers) and H3K27ac (marking active enhancers) in normal mouse bone marrow cells^28^ and H3K27ac in a MA9/Nras^G12D^ murine AML cell line (RN2)^29^ revealed that these regions represent putative poised and active enhancer elements. Regions II and III are active enhancers in both normal bone marrow and RN2 cells; however, examination of ATAC-seq signal intensity reveals greater degree of open chromatin in normal cells versus our MA9-transformed cells, consistent with down-regulation of *Mina* in tumour cells. In contrast, Region IV represents a potential poised enhancer in normal bone marrow and active enhancer in RN2 cells, consistent with the upregulation of *Crybg3* in our MA9-transformed cells. While little is known of the function or role for *Crybg3* and *Mina* in development of AML, downregulation of *Mina* and upregulation of *Crybg3* are consistent with a previous dataset examining gene expression alterations on MA9-induced transformation of GMP cells^30^. Our data identify putative enhancer regions for regulation of these and many other transcripts in bulk AML cells that could not have been captured with transcriptome analysis alone, offering new targets for therapeutic manipulation of gene expression levels.

In our analysis of the regulatory elements surrounding *Mina*, we observed a correlation between the degree of open chromatin and the transcript level. To determine whether this relationship applied genome wide, we focused on promoter regions with an ATAC-seq peak and compared the relationship between the level of chromatin accessibility at the promoter and the gene expression of that transcript. Our results demonstrate that, within a given tumour, promoter regions with the lowest chromatin accessibility level (bottom 10 and 25% of ATAC-seq peaks in terms of read counts) correspond to the progressively lowest fraction of transcripts with respect to expression level (Supplementary Fig. 6). On the other hand, promoter regions with the highest chromatin accessibility level (top 10 and 25% of ATAC-seq peaks) correspond to the most highly expressed transcripts; however, we did not observe a difference in the expression comparing the top 10% versus top 25%, suggesting an upper limit of chromatin accessibility whereby additional increases in accessibility no longer relate to increased gene expression. Altogether, these data confirm the expected overall correlations between open chromatin and gene expression patterns, but also demonstrates the qualitative differences in the information captured by these two complementary assays.

### Chromatin landscape identifies leukaemia cell of origin

**Figure 5:**
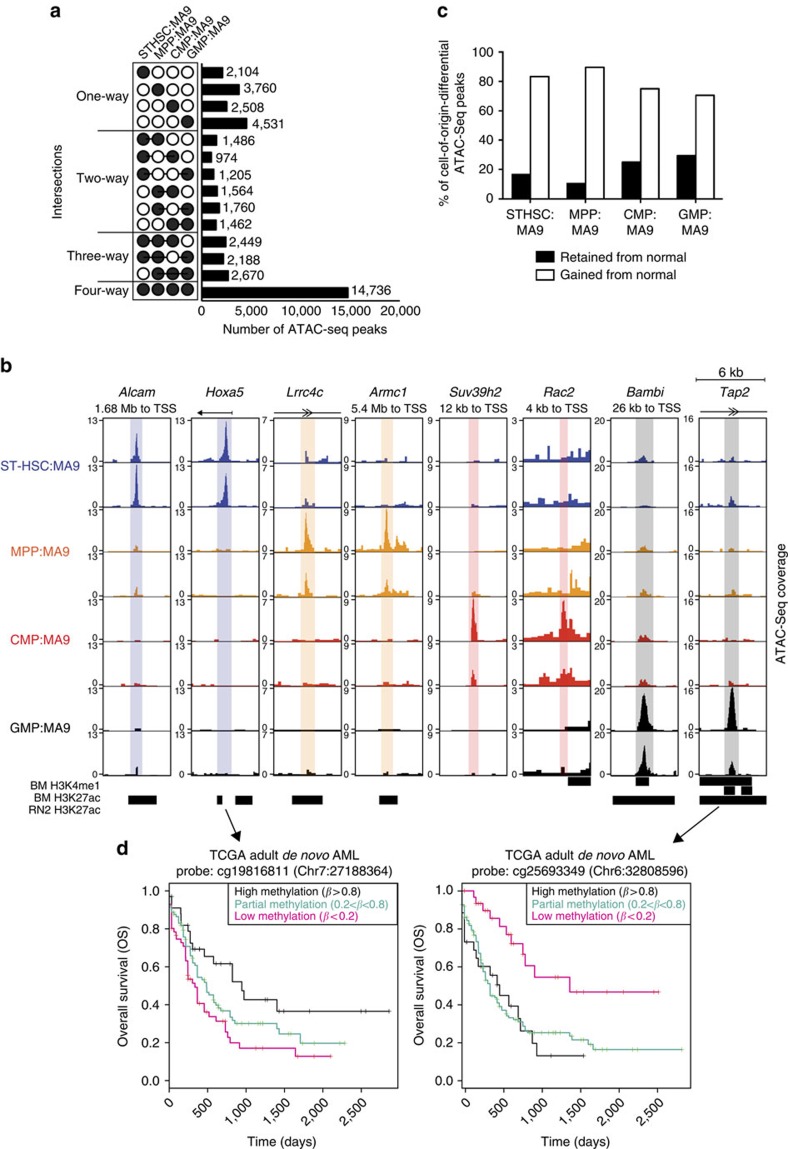
Unique open chromatin regions in bulk leukaemia cells identify AML cell of origin. (**a**) Number of intersecting ATAC-seq peaks based on leukaemia cell of origin (STHSC:MA9; *n=*2, MPP:MA9; *n*=2, CMP:MA9; *n*=2, GMP:MA9; *n*=2). Calculated numbers exclude peaks found in non-selected leukaemia subtypes. Each sample was obtained from an individual mouse. Data were collected from two biological replicate experiments. (**b**) Normalized ATAC-seq profiles of *in vivo*-derived primary bulk leukaemias from distinct cells of origin (biological replicates are shown), highlighting unique open chromatin regions around *Alcam, Hoxa5, Lrrc4c, Armc1, Suv39h2, Rac2, Bambi* and *Tap2* in 6 kb regions. Shown at bottom are H3K4me1 and H3K27ac ChIP-seq peak regions in normal murine bone marrow (BM) and H3K27ac peak regions in a MA9/Nras^G12D^ murine AML cell line (RN2). (**c**) Percentage of unique ATAC-seq peaks in each primary bulk leukaemia retained or gained from their respective normal cell of origin (STHSC:MA9; *n=*2, MPP:MA9; *n*=2, CMP:MA9; *n*=2, GMP:MA9; *n*=2). Each sample was obtained from an individual mouse. Data were collected from two biological replicate experiments. (**d**) Overall survival of human AML patients based on DNA methylation status of the CpG probe shown (*n*=200). Log-rank test; *P*=0.0170 and *P*=0.00137, respectively.

We hypothesized that unique open chromatin regions in bulk leukaemia cells may be reflective of the cell of origin, either retained during the process of transformation or uniquely gained during the process of transformation associated with differential tumour aggressiveness. Although the greatest numbers of ATAC-seq peaks were found to be common to all leukaemias, we captured many cell of origin–specific peaks (Fig. 5a). The majority of these unique regions were annotated as intergenic or intronic, suggesting that these represent regulatory regions, putative enhancers, of the genome (Supplementary Fig. 7a). To eliminate potential biases due to open chromatin coverage, differential analysis of ATAC-seq peaks between leukaemias based on cell of origin was performed after normalization for read depth within the peak regions. This analysis revealed 182 high-confidence differential peaks (Supplementary Data 2; foldchange=2.1±0.059, *P*=4.01 × 10^−5^±3.36x10^−6^, FDR=0.042±0.002). Profiling open chromatin in LSCs isolated from each of these leukaemias revealed an additional 1,420 high-confidence differential peaks based on cell of origin (Supplementary Data 3; foldchange=2.4±0.024, *P*=1.73 × 10^−4^±4.76 × 10^−6^, FDR=0.022±0.0004). Overlapping and distinct regions identified in bulk tumours versus LSCs are shown in Supplementary Fig. 7b,c). The relatively low number of overlapping regions suggests that distinct open chromatin regions may be lost and gained as cells progress from LSCs to bulk tumour cells. However, as bulk tumour sampling has greater translational potential and comparable molecular data sets from human AML are profiled at the bulk tumour level, we focused further characterization on open chromatin regions at the bulk tumour level.

To determine whether the 182 high-confidence differential peaks identified in bulk tumours represent enhancer elements, we examined H3K4me1 and/or H3K27ac ChIP-seq data from normal mouse bone marrow cells^28^, normal haematopoietic stem and progenitor cells^25^, and a MA9/Nras^G12D^ murine AML cell line (RN2)^29^. In total, we observed that 107 of these 182 peaks represent putative poised and/or active enhancers (Supplementary Data 2). As the RN2 cell line data includes an Nras^G12D^ mutation in addition to MA9, this comparison may not estimate the complete proportion of enhancers in our leukaemias. Selected examples of high-confidence differential peaks representing enhancer elements are presented with the nearest expressed gene, including *Alcam* and *Hoxa5* in STHSC:MA9, *Lrrc4c* and *Armc1* in MPP:MA9, *Suv39h2* and *Rac2* in CMP:MA9, and *Bambi* and *Tap2* in GMP:MA9 (Fig. 5b). Alterations in chromatin state in these regions were not observed to alter gene expression of the nearest coding genes (Supplementary Fig. 8a), indicating that they may be long-range *cis* enhancers^31^,^32^, or in some cases, poised regulatory elements. By comparing these regions with open chromatin regions defined by ATAC-seq of normal cells, we find that a proportion of these regions are retained from their corresponding normal cell types, including loci nearest *Alcam, Armc1, Suv39h2, Bambi* and *Tap2* (Supplementary Fig. 8b). On a global level, an average of 20.4±4.2% of tumour cell of origin–specific peaks are retained from the corresponding cell of origin (Fig. 5c). Altogether, these data demonstrate that profiling open chromatin regions of bulk tumour samples identifies unique biomarkers of leukaemia cell of origin, associated with differential leukaemia aggressiveness *in vivo*.

To determine the translational relevance of our identified loci, we lifted-over our 182 high-confidence differential peaks to the human genome (hg19) based on sequence similarity. 126 loci in hg19 were identified using this approach. Reflective of the poor sequence conservation of enhancer elements cross-species^33^, only 15 of these 126 regions were identified as enhancer elements examining the FANTOM5 database^34^,^35^ and H3K27ac ChIP-seq data from a MA9 human AML cell line (MOLM14)^36^ (Supplementary Data 2). As a comparable data set to examine our open chromatin regions (that is, genome-wide open chromatin profiling data from a large cohort of human AML patients) is currently not available, we instead utilized available DNA methylation array data from 200 *de novo* AML patients spanning the major morphologic and cytogenetic subtypes of AML^21^. It has been previously demonstrated that ∼20% of open chromatin regions genome wide show a significant inverse correlation with DNA methylation status^8^. Therefore, our rationale was that loci with low DNA methylation level in AML patient samples may correspond to our cell of origin–specific open chromatin regions. From our cell of origin–specific open chromatin peaks, examining a ±10-kb window from the centre of each peak identified overlap with 658 probes on the Infinium 450 K Human Methylation BeadChip array used in the TCGA *de novo* AML study. We found that the methylation status of 7 out of these 658 probes were significantly predictive of overall survival (Supplementary Fig. 9). For example, Chr7: 27182646-27183660 (hg19) was lifted-over from a mouse STHSC:MA9-specific open chromatin region in *Hoxa5*, where open chromatin is associated with poor outcome (Fig. 5b). In human TCGA data, low DNA methylation at Chr7: 27188364 is predictive of poor outcome in AML (Fig. 5d). Conversely, Chr6: 32801013-32801086 (hg19) was lifted-over from a mouse GMP:MA9-specific open chromatin region in *Tap2*, where open chromatin is associated with favourable outcome (Fig. 5b). In human TCGA data, low DNA methylation at Chr6: 32808596 is predictive of favourable outcome in AML (Fig. 5d). Given that only 418 probes out of >485,000 had previously been determined to be significantly associated with overall survival in the TCGA *de novo* AML data set by Cox-regression analysis^37^, our results represent a significant enrichment of predictive probes proximal to our cell of origin-specific open chromatin regions (Pearson's *χ*^2^-test; *P*<0.0001). As enhancer elements are rapidly evolving elements of the genome, we emphasize that our strategy represents a proof of principle. We anticipate experimental interrogation of open chromatin in normal human stem and progenitor cell types as well as AML patient cohorts will identify precise regions with prognostic significance based on cell of origin.

## Discussion

We have found that tumour cell of origin in MA9-driven AML significantly impacts the *in vivo* penetrance and rate of AML development, with a spectrum from HSC-derived AMLs being the most aggressive to the most differentiated progenitor cell-derived AMLs being the least aggressive. This difference in prognosis was associated with modest differences in transcriptional signatures of bulk tumours, where gene expression differences mainly distinguished stem cell-derived from progenitor cell-derived leukaemias. We hypothesized that profiling the chromatin state of regulatory regions of the genome would allow capture of regulatory elements conserved or uniquely gained during the transformation process from distinct cells of origin, reflecting transcriptional potential. Our data demonstrate that open chromatin profiling can be used as a reliable determinant of tumour cell of origin, uniquely and precisely distinguishing each cell of origin tested. This work represents a proof-of-principle to be applied to normal and transformed primary human cell types, and defines a precision medicine strategy to refine AML prognosis from bulk, circulating tumour (non-stem) cells based on the identity of the cell of origin. Furthermore, a recent report demonstrating that cell-free DNA isolated from circulating blood plasma can be used to footprint nucleosome occupancy^38^ suggesting that our proposed strategy may be more widely applicable to solid tumours using this noninvasive monitoring technique.

Work by Krivtsov *et al*.^4^ has demonstrated differential leukaemia aggressiveness initiated from HSC versus GMP, using the same MA9 model described here. Our work confirms and extends their observations, now providing data on experimentally transforming intermediate stages in the normal haematopoietic hierarchy, the MPP and CMP populations. By performing our study from staged cells of origin, our molecular signatures can be precisely and thoroughly compared with matched normal cells. Recent studies focusing on methods of identifying AML cell of origin have utilized gene expression and/or global DNA methylation data derived from the LSC subpopulation^4^,^39^,^40^. Here we also observe distinct regions of open chromatin in the LSC subpopulation that are also reflective of cell of origin, many of which are lost as these LSCs differentiate into bulk tumour cells. While pursuing these regions will have great value in furthering our understanding of LSC biology, and potentially drug resistance mechanisms, limitations in the translational context include the variability in cell surface marker expression on LSCs^41^, making it difficult to consistently sample this cell population across AML patients. Here by focusing on open chromatin regulation in bulk, circulating tumour samples, we have uncovered unique open chromatin signatures that correspond to particular cells of origin, demonstrating the utility of this strategy in determining tumour cell of origin. Moreover, this also enables us to uncover potential enhancers that may be manipulated to regulate transcriptional programs within leukaemias. Importantly, our work demonstrates that many putative enhancer elements (based on H3K4me1 and H3K27ac histone modification marks) do not alter expression of the nearest annotated transcripts, emphasizing that these may represent long-range *cis* enhancers and/or poised regulatory elements. Future work to model three-dimensional architecture within these tumour subtypes will be critical to identify the gene targets of our identified cell of origin-specific open chromatin regions. As previous work has demonstrated that AML cell of origin influences response of tumours to conventional chemotherapeutic agents^4^, we propose that open chromatin profiling may be used to predict therapeutic response in addition to disease aggressiveness.

Why are there differences in tumour aggressiveness when leukaemias are initiated from distinct cell types along a differentiation hierarchy? Gene expression profiling in our study as well as others offers some clues as to the underlying basis of differential tumour aggressiveness based on cell of origin. High expression of the oncogene *Evi1*, found in aggressive/poor prognosis AML in this study and others^4^,^12^,^13^, leads to hyperproliferation of bone marrow cells and downregulation of genes important for terminal differentiation^42^. Our gene expression data point to underlying differences in extravasation/migration, small GTPase signal transduction, and immune recognition of AML based on cell of origin. Furthermore, our data identify putative lncRNAs that may serve as novel targets for AML subtypes based on cell of origin. Altogether, this supports that multiple underlying mechanisms contribute to driving AML prognosis. Future studies mechanistically interrogating these candidate mechanisms for directed therapeutics toward AML groups, defined based on cell of origin are warranted.

Are these findings clinically relevant? Recent interrogation of human AML data from TCGA has demonstrated that cell of origin in human AML is variable^39^. Leukaemic transformation was predicted to predominantly occur at either the lymphoid-primed MPP (LMPP) or GMP stage, supporting a previous study demonstrating that human AML LSCs transcriptionally resemble LMPPs or GMPs^40^. LMPP-like LSCs were found to be the most immature^40^ and patients in this cluster are at high-cytogenetic risk^39^, while GMP-like LSCs are more mature^40^ and patients in this cluster are at low to intermediate cytogenetic risk^39^, consistent with our results demonstrating that MPP-derived AML is more aggressive *in vivo* than GMP-derived AML^40^. Interestingly, the Jung *et al*.^39^ study also identified a novel, small subset of AML patient samples that molecularly clustered with normal CMP, and a few samples clustering with normal HSC, MPP or MEP. This work suggests that a method to reliably interrogate cell of origin from bulk tumour samples, as we have identified here, will have clinical prognostic utility. The development of genome editing technology has now permitted generation of endogenous *MA9* through insertional mutagenesis in primary human haematopoietic stem and progenitor cells (HSPCs)^43^. Genome editing of prospectively isolated, normal human HSPC subpopulations will provide a valuable experimental platform to assess which chromatin loci may be retained or uniquely gained on transformation of distinct cells of origin. We propose that open chromatin profiling of bulk human AML samples, as well as normal human HSCs and progenitors, should be performed to identify the critical biomarkers with the greatest clinical relevance.

In conclusion, we have shown that open chromatin profiling reveals regulatory regions of the genome that could serve as biomarkers of tumour cell of origin, from bulk tumour sampling. Our studies provide proof of principle that the epigenetic state of the cell in which an oncogene is initially expressed contributes to the final epigenetic landscape of the tumour. Furthermore, this work defines putative enhancer regions in the mouse genome that may be manipulated to regulate gene expression within leukaemia cell subsets. Future work to identify clinically relevant enhancer regions in human AML patients based on this proof of principle will be needed to define a precision medicine strategy to determine tumour cell of origin, provide improved predictions of tumour prognosis and therapeutic response, and allow development of targeted therapies based on unique biology and heterogeneity within currently defined tumour types.

## Methods

### Experimental animals

All experiments utilized C57BL/6J female mice, between 6 and 12 weeks of age, from The Jackson Laboratory. The Jackson Laboratory's animal care and use committee (ACUC) approved all experiments.

### MA9 Retroviral Supernatant Production

Retroviral supernatant was generated as described^20^. Briefly, transient transfection of 10cm plates of HEK293T cells with 10 μg pMSCV-IRES-MLL-AF9-GFP^20^ and 10 μg π ecotropic packaging vector was performed using a Calcium Phosphate Transfection Kit (Invitrogen). After 24 h, media was replaced with fresh growth media. Retroviral supernatant was harvested 24 h later, filtered through a 0.45-μm polyvinylidene difluoride syringe filter, and utilized directly for transduction of primary cells.

### Primary cell isolation and retroviral infection

Single-cell suspensions of bone marrow were prepared by filtering crushed, pooled femurs, tibiae and iliac crests from each mouse. Bone marrow mononuclear cells were isolated by Ficoll-Paque (GE Healthcare) density centrifugation and lineage depletion was performed using the Biotin Mouse Lineage Panel (BD Biosciences), including antibodies against CD3ɛ, CD11b, B220, Gr-1 and Ter-119, with streptavidin M-280 dynabeads (Invitrogen). Lineage-depleted bone marrow cells were stained with a combination of fluorochrome-conjugated antibody clones from eBioscience or BD Biosciences: c-Kit (clone 2B8), Sca-1 (clone E13-161.7), CD150 (clone mShad150), CD34 (clone RAM34), FcγR (clone 2.462), mature lineage (Lin) marker mix (B220, CD11b, CD4, CD8, Ter-119, Gr-1) and the viability stain propidium iodide (PI). Stained cells were sorted using a FACSAria with Diva software (BD) based on the following surface marker profiles: LT-HSC (Lin^−^ Sca-1^+^ c-Kit^+^ CD150^+^ CD34^−/lo^), ST-HSC (Lin^−^ Sca-1^+^ c-Kit^+^ CD150^+^ CD34^+^), MPP (Lin^−^ Sca-1^+^ c-Kit^+^ CD150^−^ CD34^+^), CMP (Lin^−^ Sca-1^−^ c-Kit^+^ CD150^−^ CD34^+^ FcγR^lo^) and GMP (Lin^−^ Sca-1^−^ c-Kit^+^ CD150^−^ CD34^+^ FcγR^+^). Purity of sorted cells was found to be ≥95%. For transduction, cells were suspended in fresh retroviral supernatant supplemented with 10% fetal calf serum (Sigma), 100 ng ml^−1^ murine stem cell factor (mSCF), 10 ng ml^−1^ mIL-3, 10 ng ml^−1^ mIL-6 (Peprotech) and 4 μg ml^−1^ polybrene, spun at 2,500 r.p.m. for 1 h at 32 °C, then transferred to an incubator at 37 °C and 5% CO_2_. Cells were collected after 24 h and resuspended in fresh retroviral supernatant for a second spinfection, as above. 24 h after second spinfection, GFP^+^ cells were sorted by FACSAria and plated into *in vitro* culture or directly transplanted *in vivo*.

### *In vitro* culture of MA9-Transformed cells

GFP^+^ cells were plated into methocult GF M3434 (Stem Cell Technologies). After 7 days of culture, colonies were counted and pooled, and then 10^4^ cells were replated in the same medium. For the third round of culture, 5 × 10^3^ cells were plated. At the end of the third round, single colonies were plucked from methylcellulose and transferred to liquid culture containing Iscove's Modified Dulbecco's Media (IMDM) (Invitrogen) with 10% fetal calf serum (Sigma), 50 ng ml^−1^ mSCF, 10ng ml^−1^ mIL-3 and 10 ng ml^−1^ mIL-6 (Peprotech). Cultures were maintained at a density ≤1 × 10^6^ cells per ml at 37 °C and 5% CO_2_.

### *In vivo* transplantation

Recipient mice were sublethally irradiated (600 rads, ^137^Cs) and intravenously injected with 10^3^, 10^4^, or 10^5^ cells from primary leukaemia cell lines, or 500 GFP^+^ transduced primary cells.

### Analysis of leukaemic mice

Transplanted recipient mice were monitored every 4 weeks by peripheral blood sampling and differential blood count using a KX21-N Automated Hematology Analyser (Sysmex). Mice demonstrating elevated white blood cell counts and declining health status were sacrificed, and peripheral blood, spleen and bone marrow were harvested. Single-cell suspensions of peripheral blood, spleen and bone marrow were analysed by flow cytometry for GFP expression (BD FACS Calibur), and bone marrow was stained with fluorochrome-conjugated antibodies against c-Kit (clone 2B8), Sca-1 (clone E13-161.7), CD150 (clone mShad150), CD34 (clone RAM34), FcγR (clone 2.462), and mature lineage (Lin) marker mix (B220, CD11b, CD4, CD8, Ter-119 and Gr-1) to assess frequency of LSCs (L-GMP; GFP^+^ Lin^−^ Sca-1^−^ c-Kit^+^ CD150^−^ CD34^+^ FcγR^+^) using a LSRII (BD). Flow cytometry data was analysed using FlowJo software (TreeStar).

### RNA-seq library construction and analysis

50,000 bulk leukaemia (GFP^+^) cells from the spleen of recipient mice were sorted directly into 350 μl of RLT buffer (Qiagen) and flash-frozen. In addition, primary mouse ST-HSC (3,000–10,000), MPP (10,000–100,000), CMP (8,000–50,000) and GMP (10,000–50,000) were isolated as described above, sorted directly into 350 μl of RLT buffer and flash-frozen. Three to six independent biological replicates of bulk leukaemia cells and normal cells were sampled. Total RNA was isolated according to manufacturer's protocols (Qiagen) including DNase treatment, and quality was assessed using an Agilent 2100 Bioanalyzer and RNA 6000 Nano kit. RNA samples were processed using the NuGen Ovation RNA-Seq V2 kit. Amplified complementary DNA was sheared to approximately∼300 bp using a Covaris E220 Focused Ultrasonicator. RNA-seq library preparation used the TruSeq DNA sample prep kit v2 (Illumina). Sheared DNA was end repaired to generate blunt ended fragments. Magnetic bead purification was used to enrich end repaired DNA that was >100 bp. Purified fragments were ‘A' tailed and ligated to Illumina Y-adaptors containing a ‘T' overhang and unique indices for multiplexing. Ligated fragments were purified on magnetic beads followed by PCR amplification to provide Illumina flow cell compatible sequences on the ends. PCR products were purified using magnetic beads followed by QC size distribution analysis using the Agilent 2100 Bioanalyzer and the DNA 1000 chip assay. Average library size was 350 bp with insert sizes being ∼230 bp. Quantitative PCR using the Library Quantitation kit (Kapa Biosystems) was used to estimate library concentrations. Libraries were sequenced on the Illumina HiSeq 2000 platform at a sequencing depth of >35 million reads per sample. Transcript abundances were estimated for each RNA-seq sample using RSEM^44^. PCA plot to visualize similarity in transcriptome profiles was computed after log-transforming trimmed mean of M-values (TMM) normalized count data^45^. Unrooted RNA-seq dendrograms were generated from Pearson correlation coefficients derived from normalized read counts (log2 transformed) and visualized with APE as.phylo(). Resultant edge lengths were normalized for legibility. Read counts estimated for each gene by RSEM were given as input to the R package edgeR for differential expression analysis^46^. Differentially expressed genes were clustered using partition k-medioids algorithm available in the R package cluster^47^. The value of k was chosen based on the number of comparisons that resulted in clustering differentially expressed genes at a false discovery rate<5%. Clustered gene expression profiles were visualized using heatmap.2 function in R package gplots. In populations with more than 2 replicates, we chose to display the pair with the greatest correlation. Single-nucleotide variants and small insertions and deletions were identified using FreeBayes, a Bayesian genetic variant detector^22^. Variants detected using this method were filtered based on read depth and mapping quality to reduce false positive rate^23^. Germline SNPs identified in C57BL/6 mice using the Sanger Institute SNP resource^24^ and SNPs identified in any of our matched normal cell RNA-seq data were removed. To test the clinical significance of our ST-HSC-derived versus progenitor-derived gene expression signature in human AML patient data, we used TCGA gene expression signatures of 200 adult *de novo* AML patients^21^. Prognostic significance of each gene was computed by classifying TCGA samples into two groups based on expression profile of the gene. A sample with expression level higher than a threshold (median+0.5 × mad) was considered high and expression below the threshold (median−0.5 × mad) was considered low. Cox proportional hazard model was used to test the association between the expression level of the gene and clinical outcome.

### Real-time PCR

Semi-quantitative real-time PCR was performed using RT2 SYBR Green mix (Qiagen) on an ABI Biosystems 7500 and Ct values were normalized to GAPDH levels. The primers used were: *Gapdh*: 5′-CGTCCCGTAGACAAAATGGT-3′ and 5′-CTCCTGAAAGATGGTGATGG-3′, *MA9*: 5′-TGTGAAGCAGAAATGTGTGG-3′ and 5′-TGCCTTGTCACATTCACCAT-3′.

### ATAC-seq library construction and analysis

Cryopreserved leukaemic spleen and/or bone marrow samples were thawed, and FACSAria (BD) sorted 50 K bulk leukaemia (GFP+) cells or LSCs (L-GMP; GFP+ Lin- Sca-1- c-Kit+ CD34+ FcγR+). Samples were placed at 37 °C and 5% CO_2_ in IMDM plus 10% fetal calf serum for 30 min, then harvested, washed with 1 × PBS, and ATAC-seq libraries were prepared as previously described^27^. Quantitative PCR using the Library Quantitation kit (Kapa Biosystems) was used to estimate library concentrations. Libraries were sequenced on the Illumina HiSeq 2000 platform generating 2 × 150 bp paired end reads at a sequencing depth of ∼30–50 million reads per sample. Reads were aligned to the mouse mm9 assembly using bowtie2. Only reads that uniquely mapped to the genome were used in subsequent analysis. Duplicate reads were eliminated to avoid potential PCR amplification artifacts and to eliminate mitochondrial DNA duplicates. Post-alignment filtering resulted in ∼20–30 million uniquely aligned singleton reads per library. Samples with <10 million uniquely aligned reads were considered noisy and were excluded from the analysis. ATAC-seq peaks in each sample were identified using MACS2 48 with the following settings: MACS2-2.1.0.20140616/bin/macs2 callpeak -t <input tag file> -f BED -n <output peak file> -g 'mm' --nomodel --shift -100 --extsize 200 -B –broad. Peak annotation was performed via HOMER v4.6 annotatePeaks.pl for the mm9 assembly. Previously published ATAC-seq libraries from LSK, CMP and GMP populations were downloaded from GEO (GEO: GSE59992)^25^ and processed as described above. Heatmap presentation of cell type correlation was computed via Pearson correlation matrix of HOMER peak scores, following bedtools intersect replicate merging and visualized with gplots heatmap.2(). ATAC-seq data were visualized on the UCSC genome browser^49^ (http://genome.ucsc.edu/) with mouse mm9 assembly, along with bed files from ChIP-seq analysis of H3K4me1 and H3K27ac in mouse C57BL/6 bone marrow (ENCODE)^28^ and H3K27ac in a MA9/Nras^G12D^ murine AML cell line (RN2) (GEO GSM1262348)^29^. RNA-seq data subset on promoter ATAC-seq peak score utilized log2(FPKM+1) and was generated by edcf() function.

### Analysis of human AML enhancers and DNA methylation

Lift-over of cell of origin–specific open chromatin peaks from the mouse (mm9) to the human (hg19) genome was performed using UCSC liftOver tool. These loci were assessed for enhancer potential by comparison with the FANTOM5 enhancer database^34^,^35^ and H3K27ac ChIP-seq data of the human MA9 cell line MOLM14 (GEO GSM1587893)^36^ using Bedtools. To assess prognostic significance of DNA methylation status surrounding these loci, regions of ±10 kb from the centre of each open chromatin peak were examined. All CpG probes on the Infinium 450 K Human Methylation BeadChip array that fell within these regions were tested individually for prognostic significance in human AML TCGA data^21^. Methylation status of each sample, for each probe, was classified as high (*β*>0.8), partial (0.2<*β*<0.8) or low (*β*<0.2). Cox proportional hazard model was used to test association of methylation status and clinical outcome.

### Statistical analyses

For overall survival, log-rank (Mantel-Cox) test was performed on Kaplan–Meier survival curves. A minimum of five mice per sample type and cell dose was chosen to detect a hazards ratio of >3 with 80% power at an error rate of 5%. On the basis of pre-established criteria, animals were excluded from the analysis if they did not demonstrate >1% detectable GFP^+^ cells in the peripheral blood by 1 month post-transplant. Recipient mice were randomized for which cell line or primary AML cell type they were transplanted with. Analysis of mice was not blinded during the experiment. Limiting dilution analysis was performed with ELDA software^50^. Statistical analysis of non-survival data was performed by non-parametric one-way analysis of variance (Kruskal–Wallis test) followed by Dunn's multiple comparisons test, or two-way analysis of variance followed by Tukey's multiple comparisons test. For these experiments, a minimum of 3 samples per condition was chosen to detect statistical differences with >80% power at an error rate of 5%. Variance was similar between the groups that were statistically compared. Pearson's correlation coefficient calculation was used to calculate overall similarity between RNA-seq transcriptomes or ATAC-seq global open chromatin signatures. Pearson's *χ*^2^-tests were used for determining significance of the enrichment of prognostic data from published TCGA human *de novo* AML data.

### Data availability

RNA-seq and ATAC-seq data can be found in GEO under accession number GSE74691. All other relevant code is available from the authors on request.

## Additional information

**How to cite this article**: George, J. *et al.* Leukaemia cell of origin identified by chromatin landscape of bulk tumour cells. *Nat. Commun.* 7:12166 doi: 10.1038/ncomms12166 (2016).

## Supplementary Material

Supplementary Information Supplementary Figures 1-9 and Supplementary Tables 1-5.

Supplementary Data 1 Normalized counts of differentially expressed genes between at least two leukemia subtypes based on cell-of-origin (fold change > 2, FDR < 0.05)

Supplementary Data 2 Differential ATAC-seq peaks in bulk leukemia cells based on leukemia cell-of-origin Presence of ChIP-seq peak (H3K4me1 and/or H3K27ac) in mouse and human normal and leukemia cells are shown as shaded boxes. Human (hg19) genomic coordinates corresponding to differential peaks are shown.

Supplementary Data 3 Differential ATAC-seq peaks in leukemia stem cells based on leukemia cell-of-origin Overlapping regions also identified in bulk leukemia cells are shown in bold font. Presence of ChIP-seq peak (H3K27ac) in mouse leukemia cells is shown as shaded box.
