# Supplementary material for: Leukaemia cell of origin identified by chromatin landscape of bulk tumour cells
Source: Nat Commun. 2016 Jul 11;7:12166. doi: 10.1038/ncomms12166 (PMC4942573; doi:10.1038/ncomms12166)
Supplement: Supplementary Information — Supplementary Figures 1-9 and Supplementary Tables 1-5. [file ncomms12166-s1.pdf]

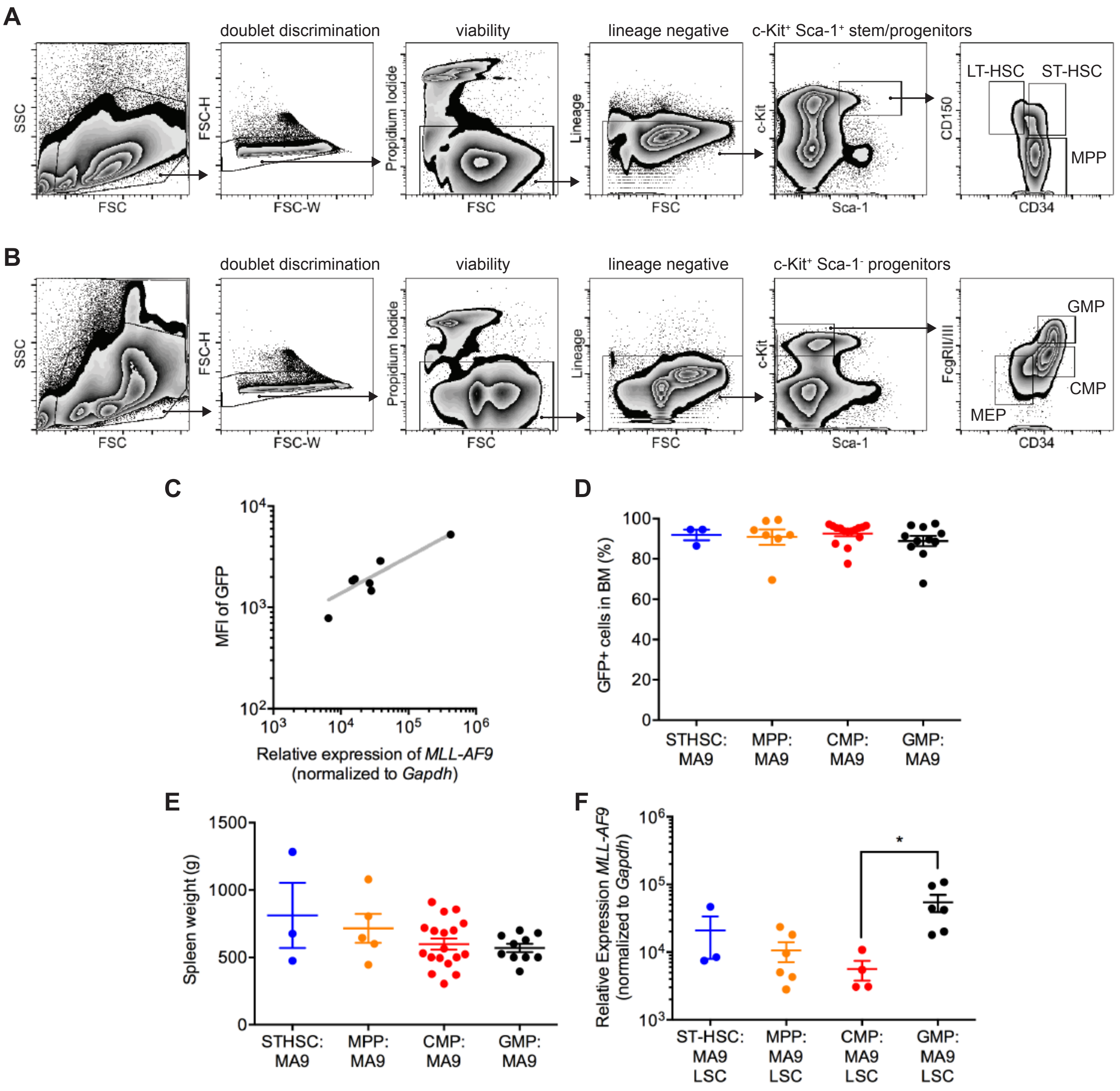

Supplementary Figure 1. Similar phenotypes of *in vivo*-derived MLL-AF9 AML from distinct cells-of-origin (A) Flow cytometry (FACS) gating strategy for isolation of LT-HSC, ST-HSC and MPP. (B) FACS gating strategy for isolation of CMP, GMP and MEP. (C) Correlation between MFI of GFP in bulk tumor cells with *MLL-AF9* relative expression measured by real-time PCR (n = 7 biological replicates obtained from individual mice). Pearson r = 0.9256, P = 0.0028. (D) Frequency of GFP^+ cells in BM of mice with *in vivo*-derived AML from distinct cells-of-origin (STHSC:MA9; n = 3, MPP:MA9; n = 7, CMP:MA9; n = 15, GMP:MA9; n = 11). Data were collected over two biological replicate experiments. Center bars indicate mean. Error bars indicate s.e.m. Kruskal-Wallis test; P = 0.6304. (E) Average spleen weights of terminal mice with AML from distinct cells-of-origin (STHSC:MA9; n = 3, MPP:MA9; n = 5, CMP:MA9; n = 18, GMP:MA9; n = 10). Data were collected over two biological replicate experiments. Center bars indicate mean. Error bars indicate s.e.m. Kruskal-Wallis test; P = 0.7260. (F) Relative expression of *MLL-AF9* assessed by real-time PCR in LSCs (STHSC:MA9; n = 3, MPP:MA9; n = 6, CMP:MA9; n = 4, GMP:MA9; n = 6). Data were collected over two biological replicate experiments. Center bars indicate mean. Error bars indicate s.e.m. Kruskal-Wallis test; P = 0.0106. Dunn's multiple comparisons test; * P < 0.05.

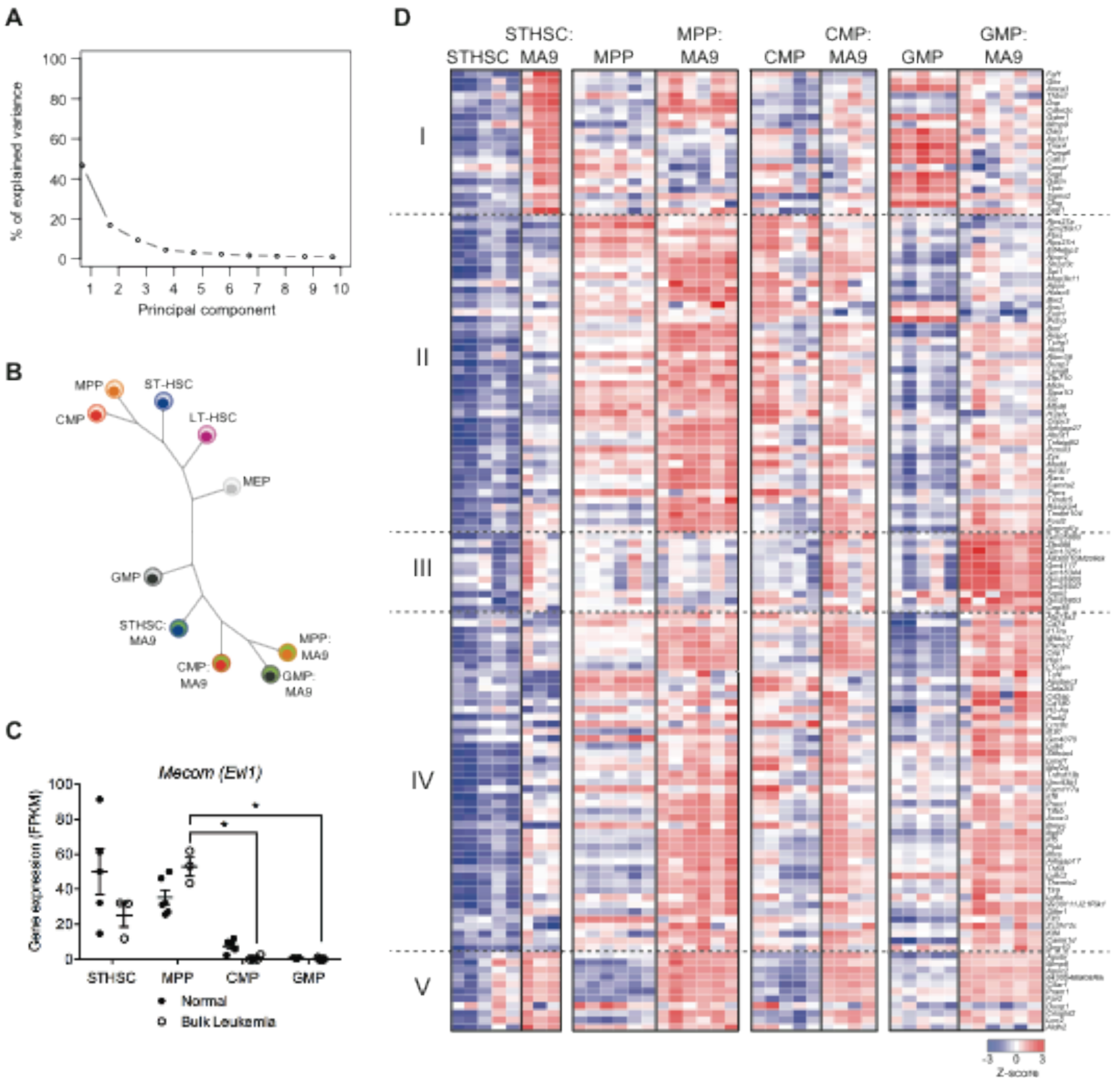

Supplementary Figure 2. **RNA-seq analysis of MLL-AF9-driven leukemias based on cell-of-origin** (A) Scree plot of eigenvalues from RNA-seq principal component analysis. (B) Hierarchical clustering of leukemias derived from distinct cells-of-origin and normal cellular counterparts based on RNA-seq transcriptomes (LT-HSC; *n* = 5, ST-HSC; *n* = 5, MPP; *n* = 6, CMP; *n* = 5, GMP; *n* = 5, MEP; *n* = 4, STHSC:MA9; *n* = 3, MPP:MA9; *n* = 6, CMP:MA9, *n* = 4, GMP:MA9, *n* = 6). Each sample was obtained from an individual mouse. Data were collected from two biological replicate experiments. (C) RNA-seq FPKM of *Evi1* in leukemias from distinct cells-of-origin and normal cellular counterparts (ST-HSC; *n* = 5, MPP; *n* = 6, CMP; *n* = 5, GMP; *n* = 5, STHSC:MA9; *n* = 3, MPP:MA9; *n* = 3, CMP:MA9, *n* = 4, GMP:MA9, *n* = 5). Each sample was obtained from an individual mouse. Data were collected from two biological replicate experiments. Center bars indicate mean. Error bars indicate s.e.m. Kruskal-Wallis test; $P < 0.0001$. Dunn's multiple comparisons test; * $P < 0.05$. (D) K-medioids clustering (K=5) of 133 differentially expressed genes (fold change > 2, $FDR < 0.05$) in leukemias based on cell-of-origin as well as matched normal cellular counterparts (ST-HSC; *n* = 5, STHSC:MA9; *n* = 3, MPP; *n* = 6, MPP:MA9; *n* = 6, CMP; *n* = 5, CMP:MA9, *n* = 4, GMP; *n* = 5, GMP:MA9, *n* = 6). Each sample was obtained from an individual mouse. Data were collected from two biological replicate experiments.

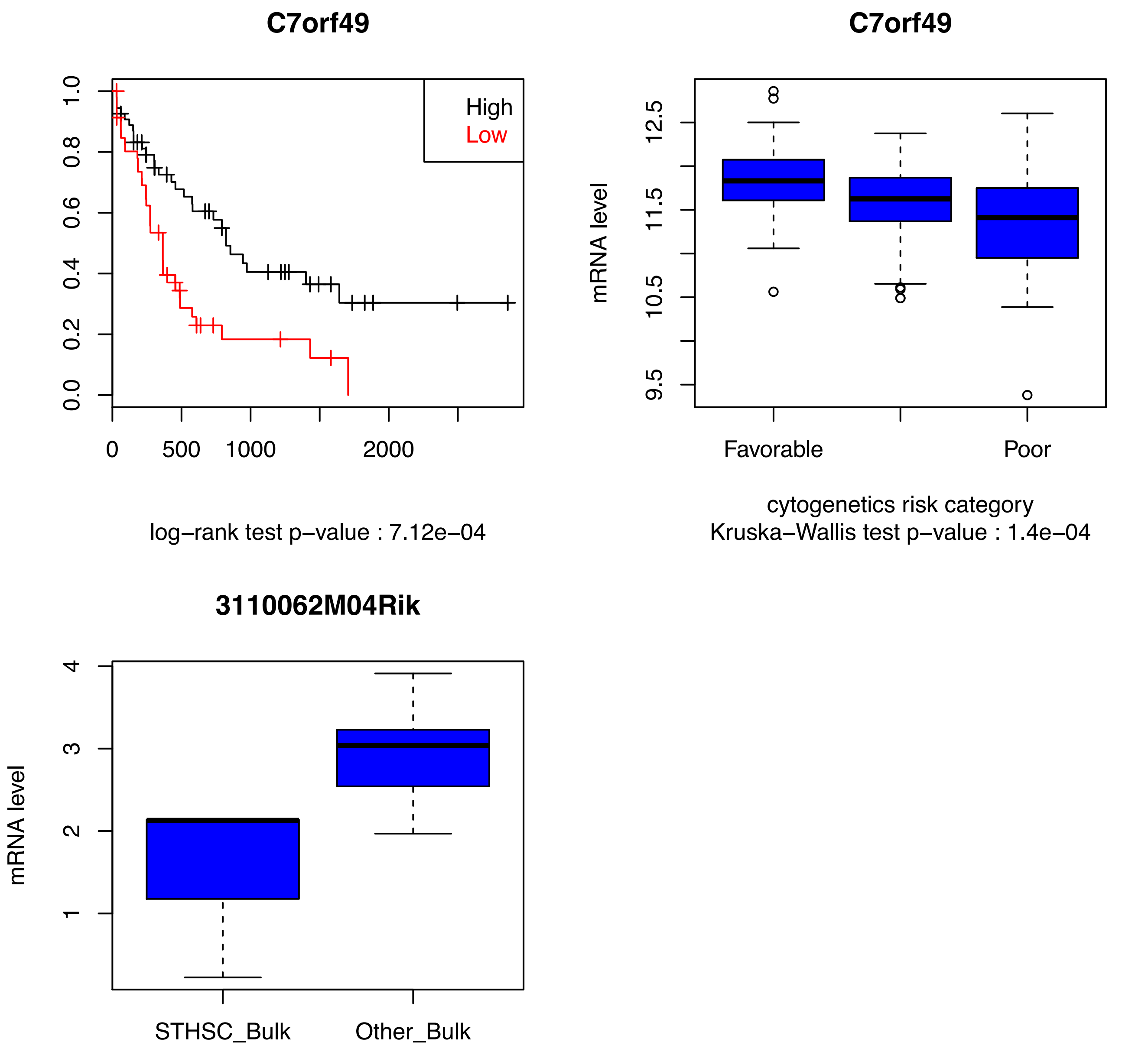

Supplementary Figure 3. Differentially expressed genes in HSC-derived AML versus progenitor-derived AML are predictive of outcome in human AML (top left panel) Kaplan-Meier analysis of overall survival of human AML patients with high and low expression of the annotated gene. P-values were calculated using log-rank test. (top right panel) Boxplot of mRNA expression level of annotated gene in AML patients with favorable, intermediate and poor cytogenetic-risk. P-values were calculated using the Kruskal-Wallis test. (lower left panel) Boxplot of mRNA expression level in STHSC:MA9 bulk leukemias versus other MA9 bulk tumors (MPP:MA9, CMP:MA9, GMP:MA9).

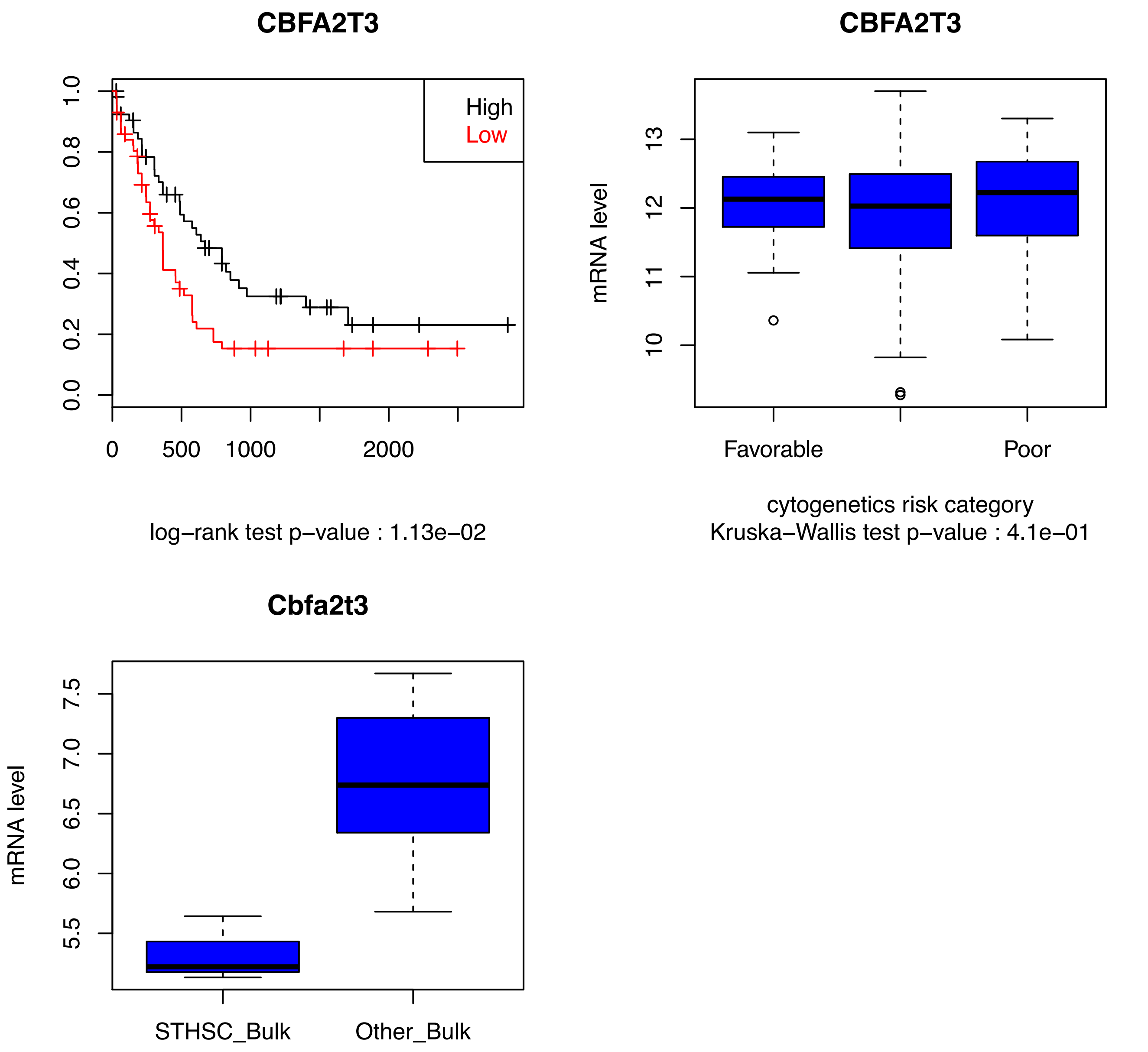

Supplementary Figure 3 (cont'd). Differentially expressed genes in HSC-derived AML versus progenitor-derived AML are predictive of outcome in human AML (top left panel) Kaplan-Meier analysis of overall survival of human AML patients with high and low expression of the annotated gene. P-values were calculated using log-rank test. (top right panel) Boxplot of mRNA expression level of annotated gene in AML patients with favorable, intermediate and poor cytogenetic-risk. P-values were calculated using the Kruskal-Wallis test. (lower left panel) Boxplot of mRNA expression level in STHSC:MA9 bulk leukemias versus other MA9 bulk tumors (MPP:MA9, CMP:MA9, GMP:MA9).

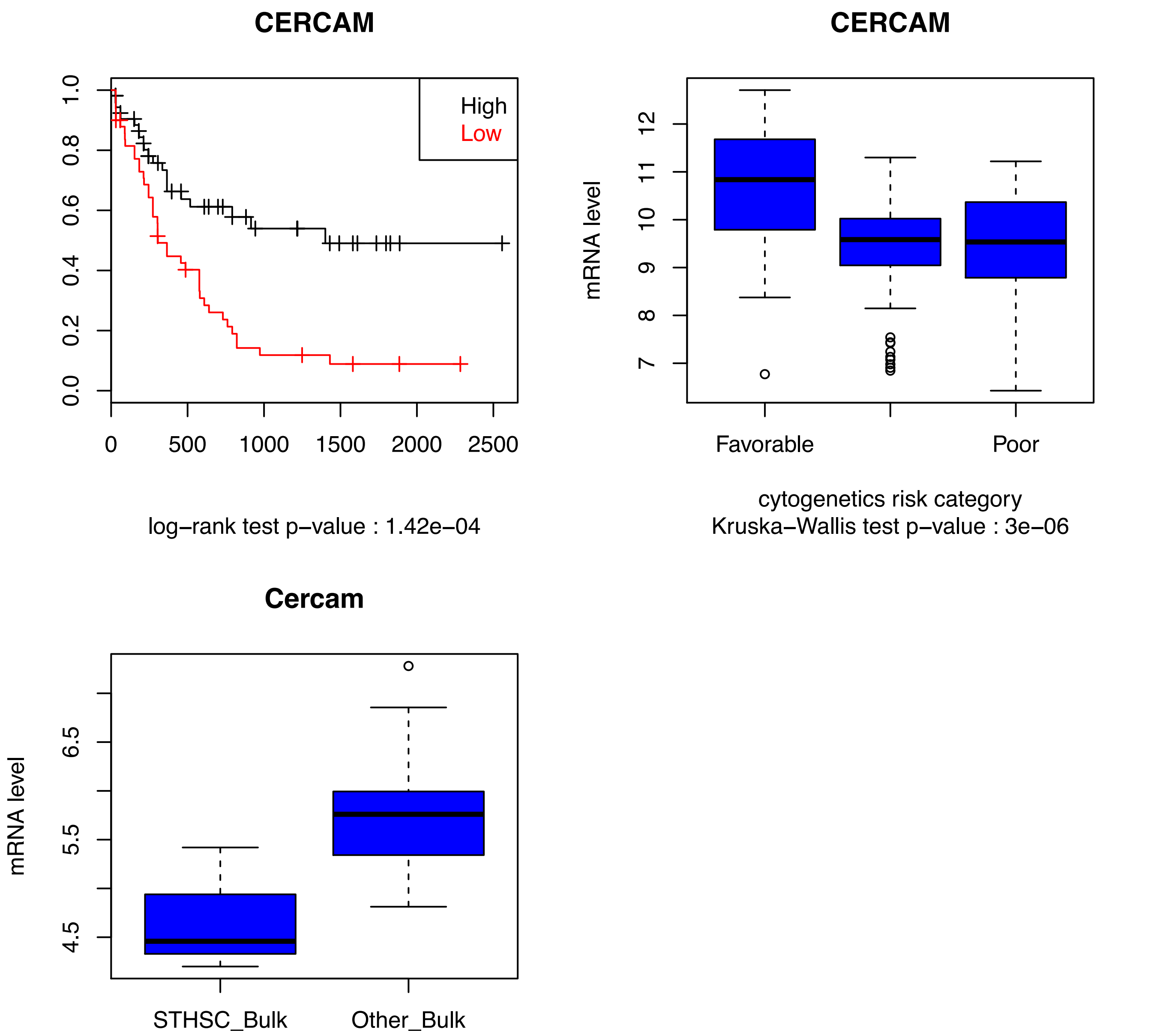

Supplementary Figure 3 (cont'd). Differentially expressed genes in HSC-derived AML versus progenitor-derived AML are predictive of outcome in human AML (top left panel) Kaplan-Meier analysis of overall survival of human AML patients with high and low expression of the annotated gene. P-values were calculated using log-rank test. (top right panel) Boxplot of mRNA expression level of annotated gene in AML patients with favorable, intermediate and poor cytogenetic-risk. P-values were calculated using the Kruskal-Wallis test. (lower left panel) Boxplot of mRNA expression level in STHSC:MA9 bulk leukemias versus other MA9 bulk tumors (MPP:MA9, CMP:MA9, GMP:MA9).

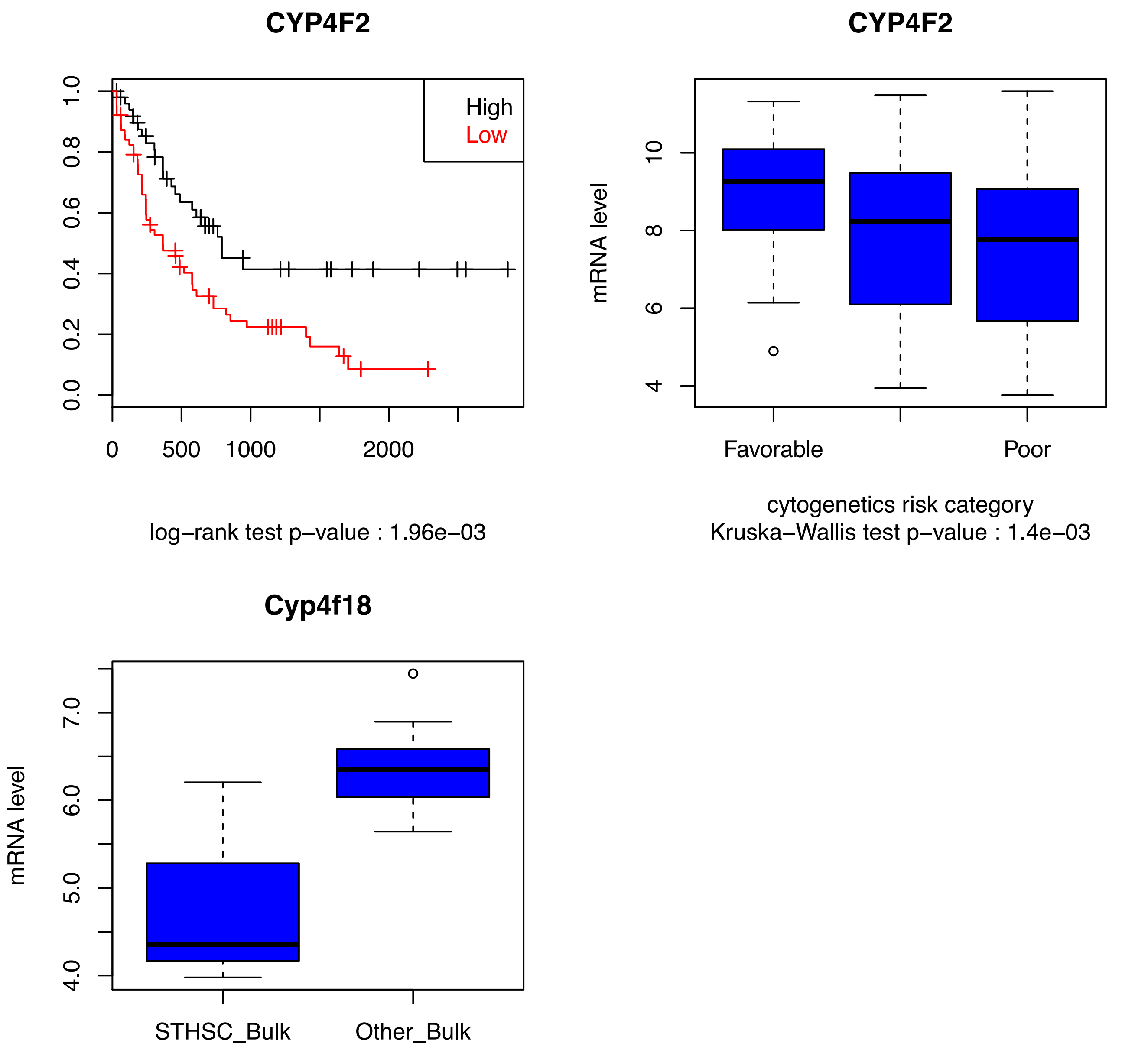

Supplementary Figure 3 (cont'd). Differentially expressed genes in HSC-derived AML versus progenitor-derived AML are predictive of outcome in human AML (top left panel) Kaplan-Meier analysis of overall survival of human AML patients with high and low expression of the annotated gene. P-values were calculated using log-rank test. (top right panel) Boxplot of mRNA expression level of annotated gene in AML patients with favorable, intermediate and poor cytogenetic-risk. P-values were calculated using the Kruskal-Wallis test. (lower left panel) Boxplot of mRNA expression level in STHSC:MA9 bulk leukemias versus other MA9 bulk tumors (MPP:MA9, CMP:MA9, GMP:MA9).

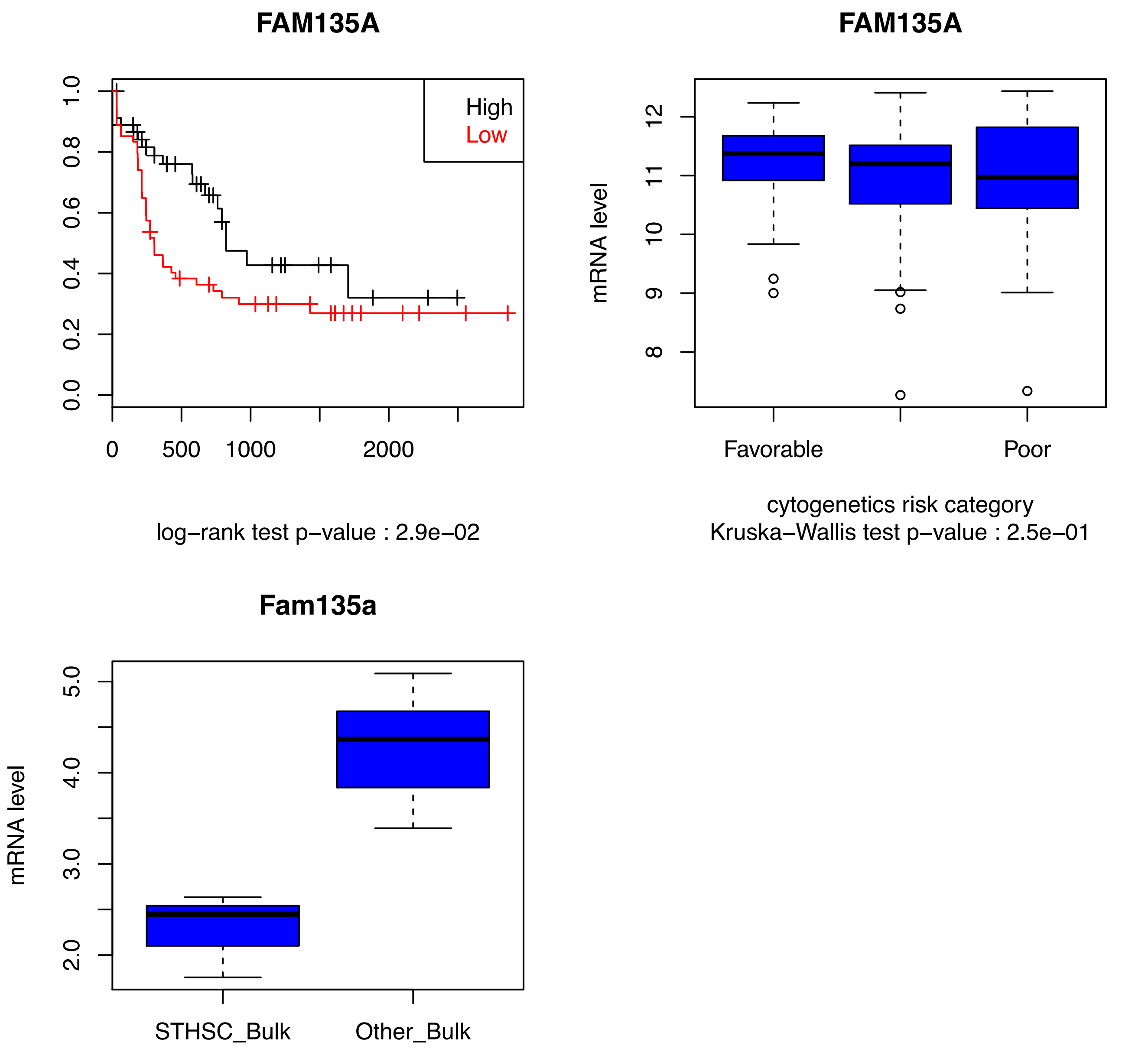

Supplementary Figure 3 (cont'd). Differentially expressed genes in HSC-derived AML versus progenitor-derived AML are predictive of outcome in human AML (top left panel) Kaplan-Meier analysis of overall survival of human AML patients with high and low expression of the annotated gene. P-values were calculated using log-rank test. (top right panel) Boxplot of mRNA expression level of annotated gene in AML patients with favorable, intermediate and poor cytogenetic-risk. P-values were calculated using the Kruskal-Wallis test. (lower left panel) Boxplot of mRNA expression level in STHSC:MA9 bulk leukemias versus other MA9 bulk tumors (MPP:MA9, CMP:MA9, GMP:MA9).

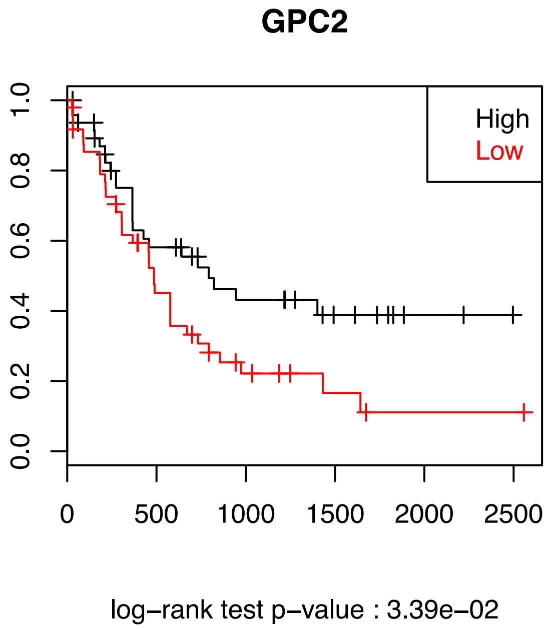

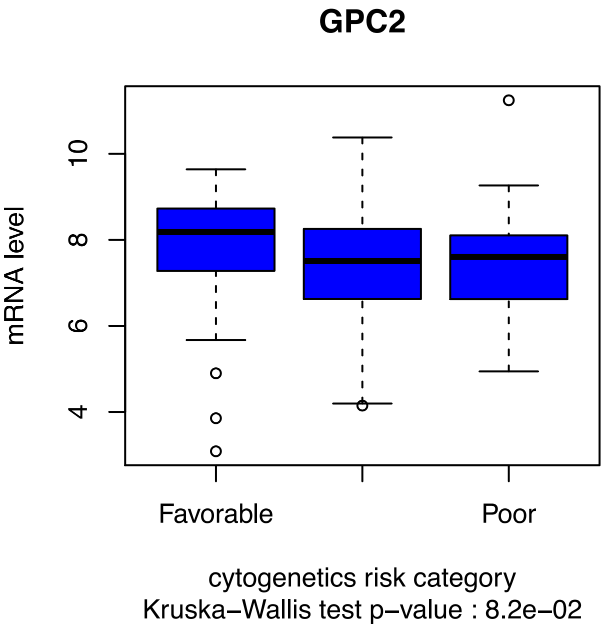

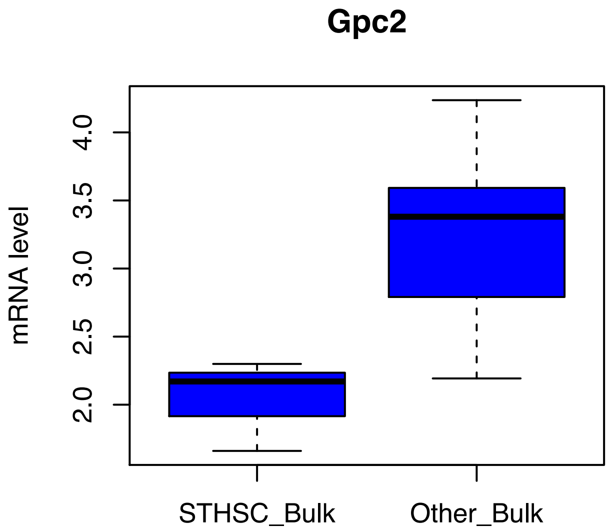

Supplementary Figure 3 (cont'd). Differentially expressed genes in HSC-derived AML versus progenitor-derived AML are predictive of outcome in human AML (top left panel) Kaplan-Meier analysis of overall survival of human AML patients with high and low expression of the annotated gene. P-values were calculated using log-rank test. (top right panel) Boxplot of mRNA expression level of annotated gene in AML patients with favorable, intermediate and poor cytogenetic-risk. P-values were calculated using the Kruskal-Wallis test. (lower left panel) Boxplot of mRNA expression level in STHSC:MA9 bulk leukemias versus other MA9 bulk tumors (MPP:MA9, CMP:MA9, GMP:MA9).

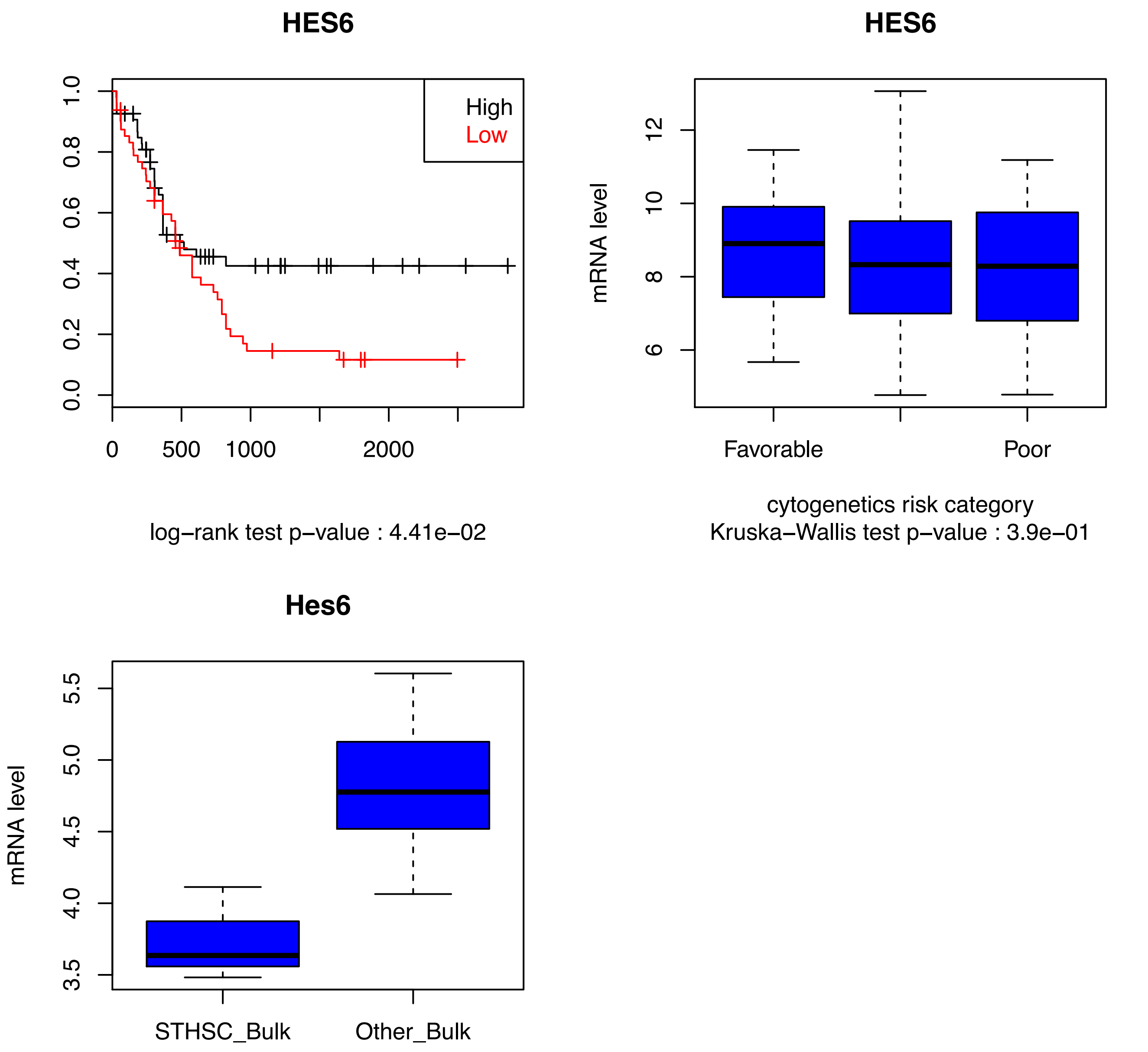

Supplementary Figure 3 (cont'd). Differentially expressed genes in HSC-derived AML versus progenitor-derived AML are predictive of outcome in human AML (top left panel) Kaplan-Meier analysis of overall survival of human AML patients with high and low expression of the annotated gene. P-values were calculated using log-rank test. (top right panel) Boxplot of mRNA expression level of annotated gene in AML patients with favorable, intermediate and poor cytogenetic-risk. P-values were calculated using the Kruskal-Wallis test. (lower left panel) Boxplot of mRNA expression level in STHSC:MA9 bulk leukemias versus other MA9 bulk tumors (MPP:MA9, CMP:MA9, GMP:MA9).

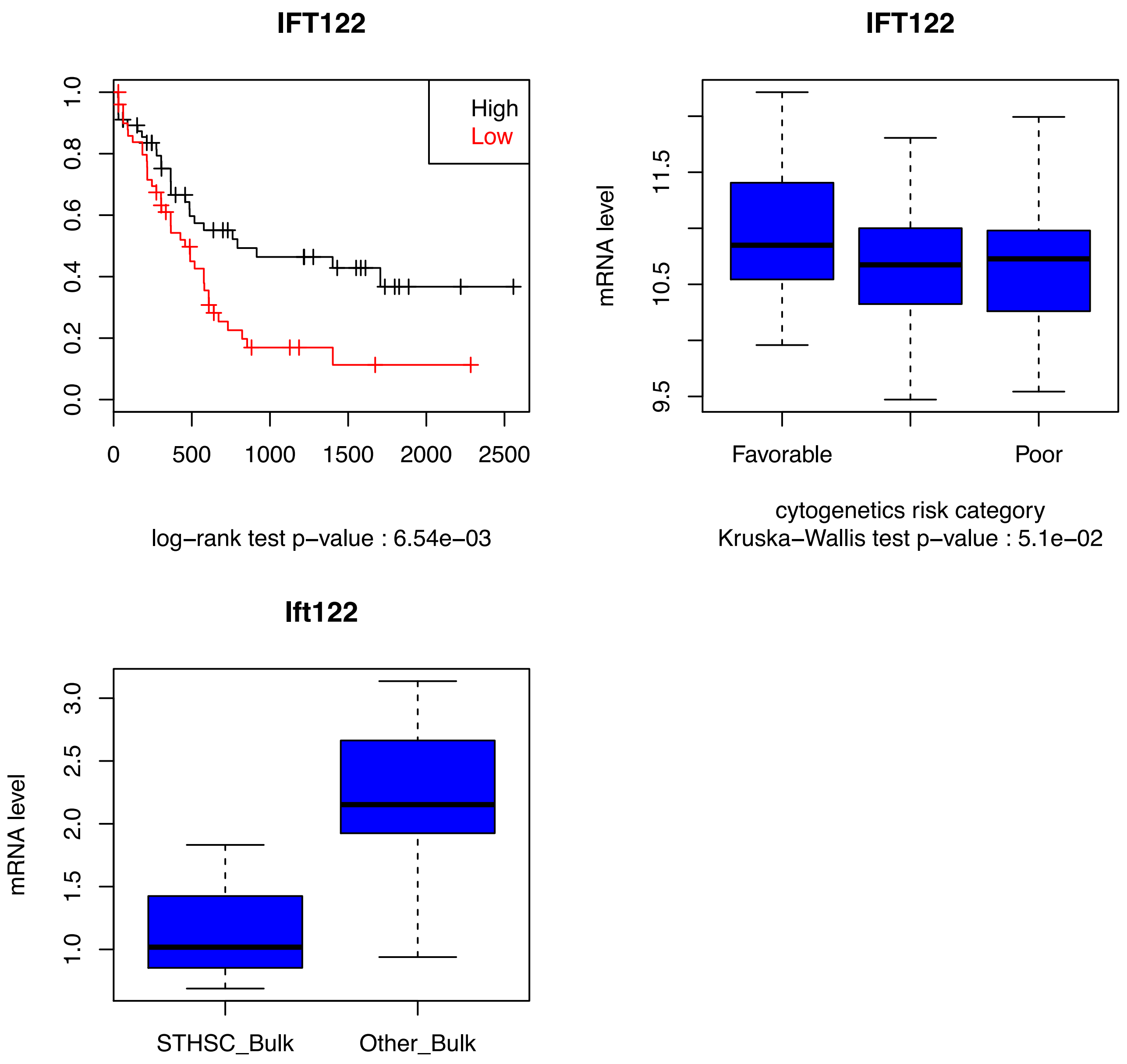

Supplementary Figure 3 (cont'd). Differentially expressed genes in HSC-derived AML versus progenitor-derived AML are predictive of outcome in human AML (top left panel) Kaplan-Meier analysis of overall survival of human AML patients with high and low expression of the annotated gene. P-values were calculated using log-rank test. (top right panel) Boxplot of mRNA expression level of annotated gene in AML patients with favorable, intermediate and poor cytogenetic-risk. P-values were calculated using the Kruskal-Wallis test. (lower left panel) Boxplot of mRNA expression level in STHSC:MA9 bulk leukemias versus other MA9 bulk tumors (MPP:MA9, CMP:MA9, GMP:MA9).

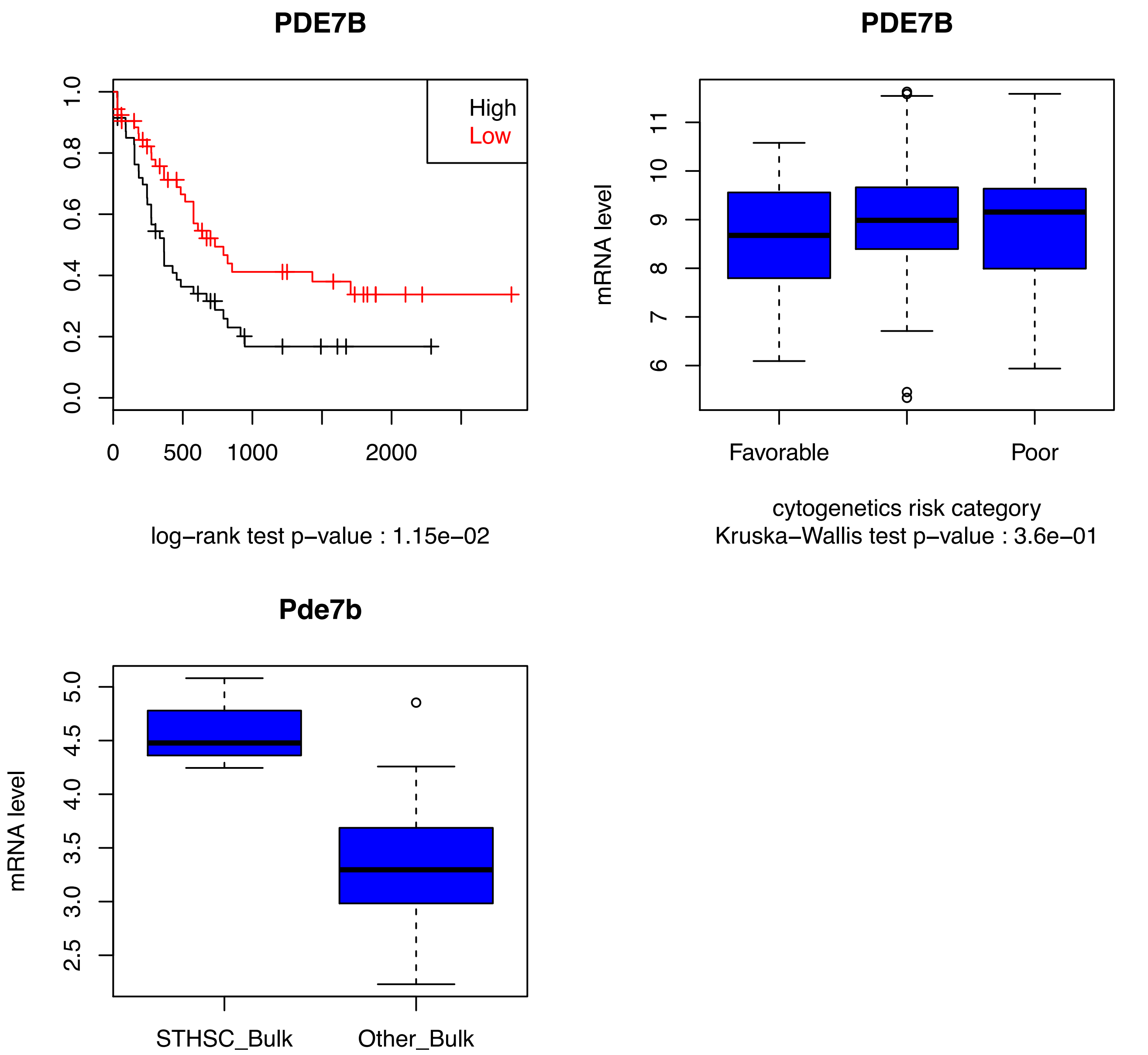

Supplementary Figure 3 (cont'd). Differentially expressed genes in HSC-derived AML versus progenitor-derived AML are predictive of outcome in human AML (top left panel) Kaplan-Meier analysis of overall survival of human AML patients with high and low expression of the annotated gene. P-values were calculated using log-rank test. (top right panel) Boxplot of mRNA expression level of annotated gene in AML patients with favorable, intermediate and poor cytogenetic-risk. P-values were calculated using the Kruskal-Wallis test. (lower left panel) Boxplot of mRNA expression level in STHSC:MA9 bulk leukemias versus other MA9 bulk tumors (MPP:MA9, CMP:MA9, GMP:MA9).

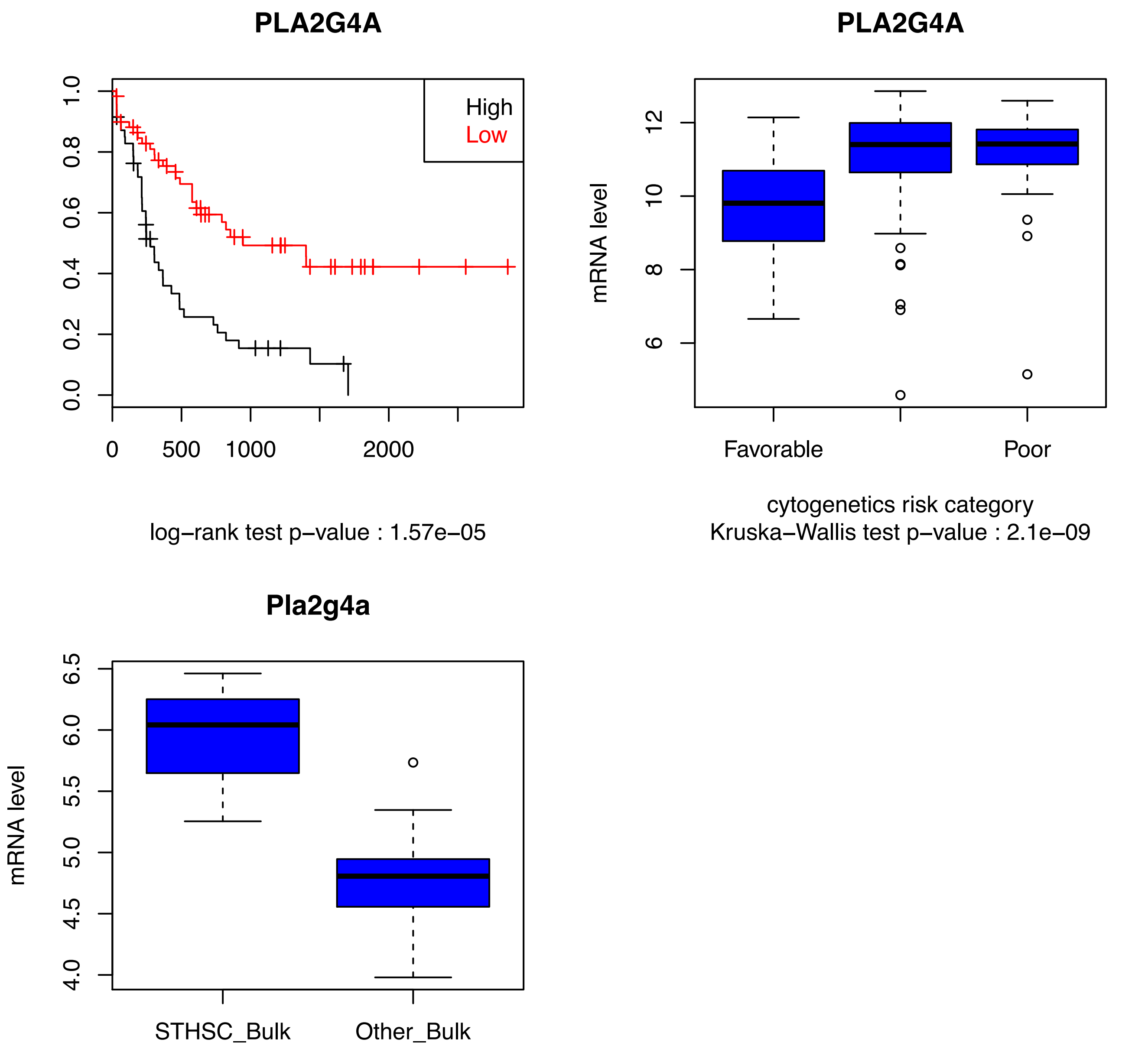

Supplementary Figure 3 (cont'd). Differentially expressed genes in HSC-derived AML versus progenitor-derived AML are predictive of outcome in human AML (top left panel) Kaplan-Meier analysis of overall survival of human AML patients with high and low expression of the annotated gene. P-values were calculated using log-rank test. (top right panel) Boxplot of mRNA expression level of annotated gene in AML patients with favorable, intermediate and poor cytogenetic-risk. P-values were calculated using the Kruskal-Wallis test. (lower left panel) Boxplot of mRNA expression level in STHSC:MA9 bulk leukemias versus other MA9 bulk tumors (MPP:MA9, CMP:MA9, GMP:MA9).

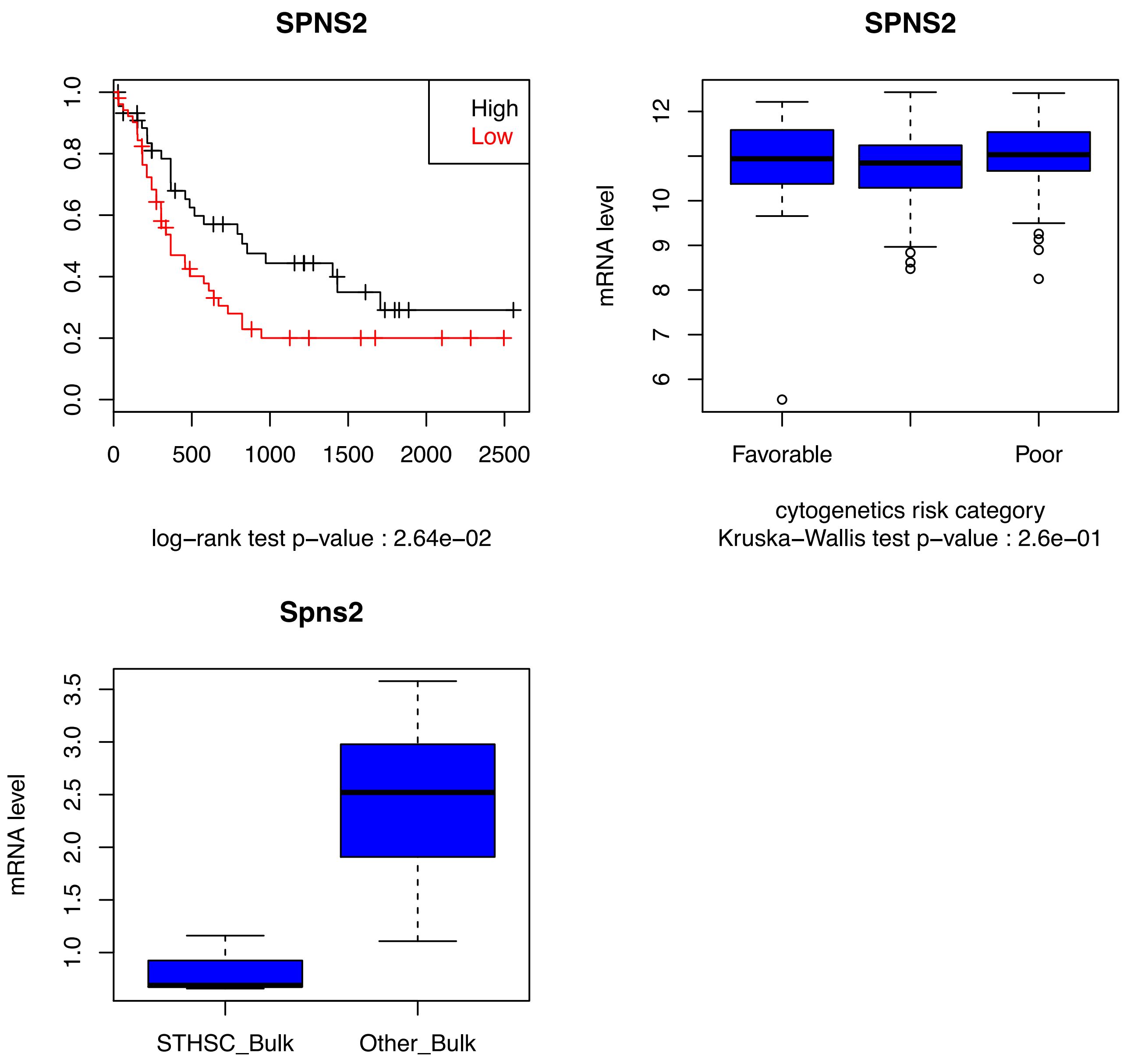

Supplementary Figure 3 (cont'd). Differentially expressed genes in HSC-derived AML versus progenitor-derived AML are predictive of outcome in human AML (top left panel) Kaplan-Meier analysis of overall survival of human AML patients with high and low expression of the annotated gene. P-values were calculated using log-rank test. (top right panel) Boxplot of mRNA expression level of annotated gene in AML patients with favorable, intermediate and poor cytogenetic-risk. P-values were calculated using the Kruskal-Wallis test. (lower left panel) Boxplot of mRNA expression level in STHSC:MA9 bulk leukemias versus other MA9 bulk tumors (MPP:MA9, CMP:MA9, GMP:MA9).

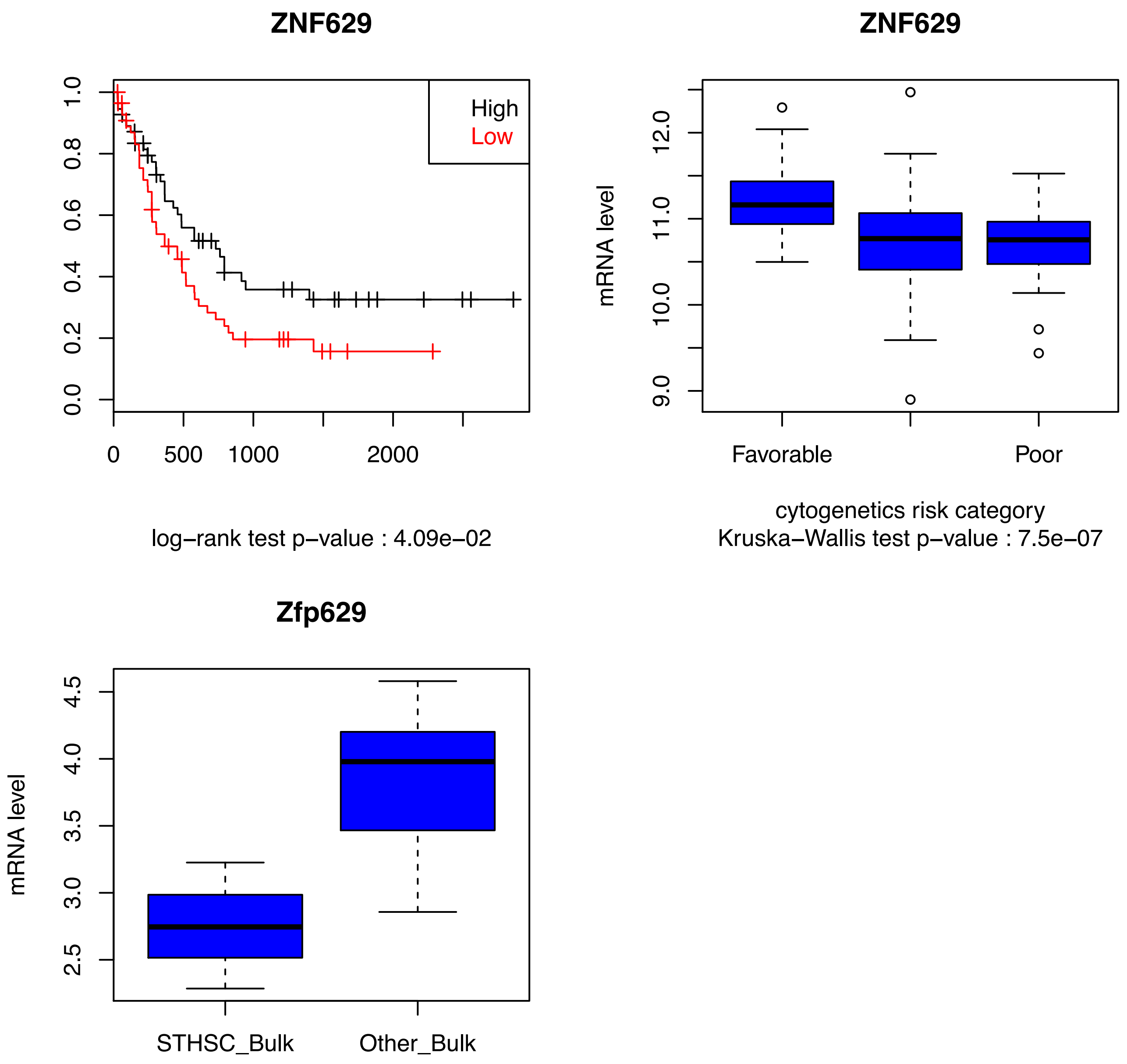

Supplementary Figure 3 (cont'd). Differentially expressed genes in HSC-derived AML versus progenitor-derived AML are predictive of outcome in human AML (top left panel) Kaplan-Meier analysis of overall survival of human AML patients with high and low expression of the annotated gene. P-values were calculated using log-rank test. (top right panel) Boxplot of mRNA expression level of annotated gene in AML patients with favorable, intermediate and poor cytogenetic-risk. P-values were calculated using the Kruskal-Wallis test. (lower left panel) Boxplot of mRNA expression level in STHSC:MA9 bulk leukemias versus other MA9 bulk tumors (MPP:MA9, CMP:MA9, GMP:MA9).

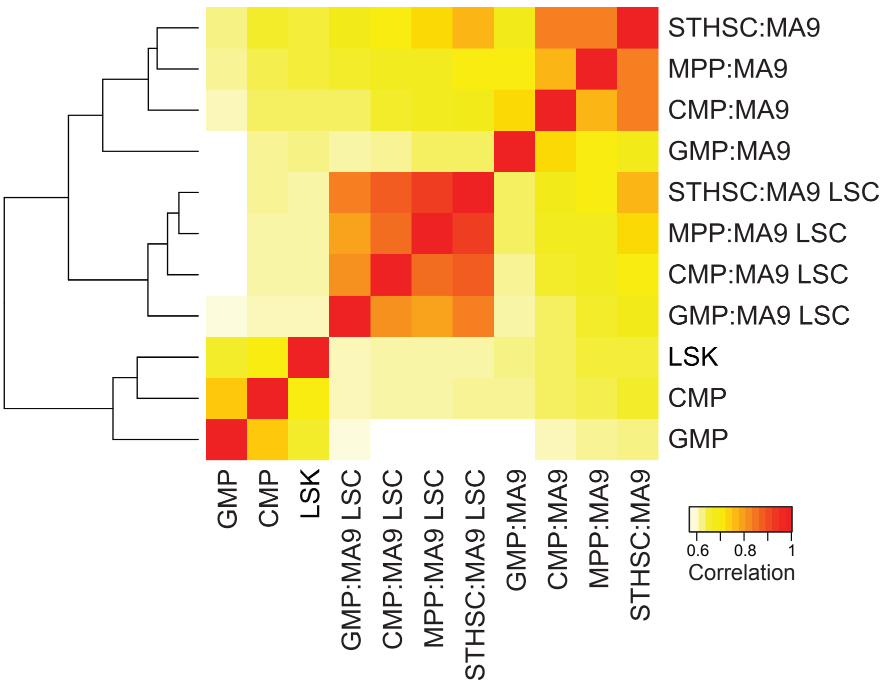

Supplementary Figure 4. ATAC-seq profiles of leukemia stem cells versus bulk leukemia cells
Heatmap of Pearson correlation analysis of open chromatin regions in LSCs derived from distinct cells-of-origin and their normal cellular counterparts (LSK; *n* = 3, CMP; *n* = 3, GMP; *n* = 2, STHSC:MA9; *n* = 2, MPP:MA9; *n* = 2, CMP:MA9; *n* = 2, GMP:MA9; *n* = 2, STHSC:MA9 LSC; *n* = 1, MPP:MA9 LSC; *n* = 1, CMP:MA9 LSC; *n* = 1, GMP:MA9 LSC; *n* = 1). LSK; Lin^- $Sca\text{-}1^+$ $c\text{-}Kit^+$, includes ST-HSC and MPP cells. Each sample was obtained from an individual mouse.

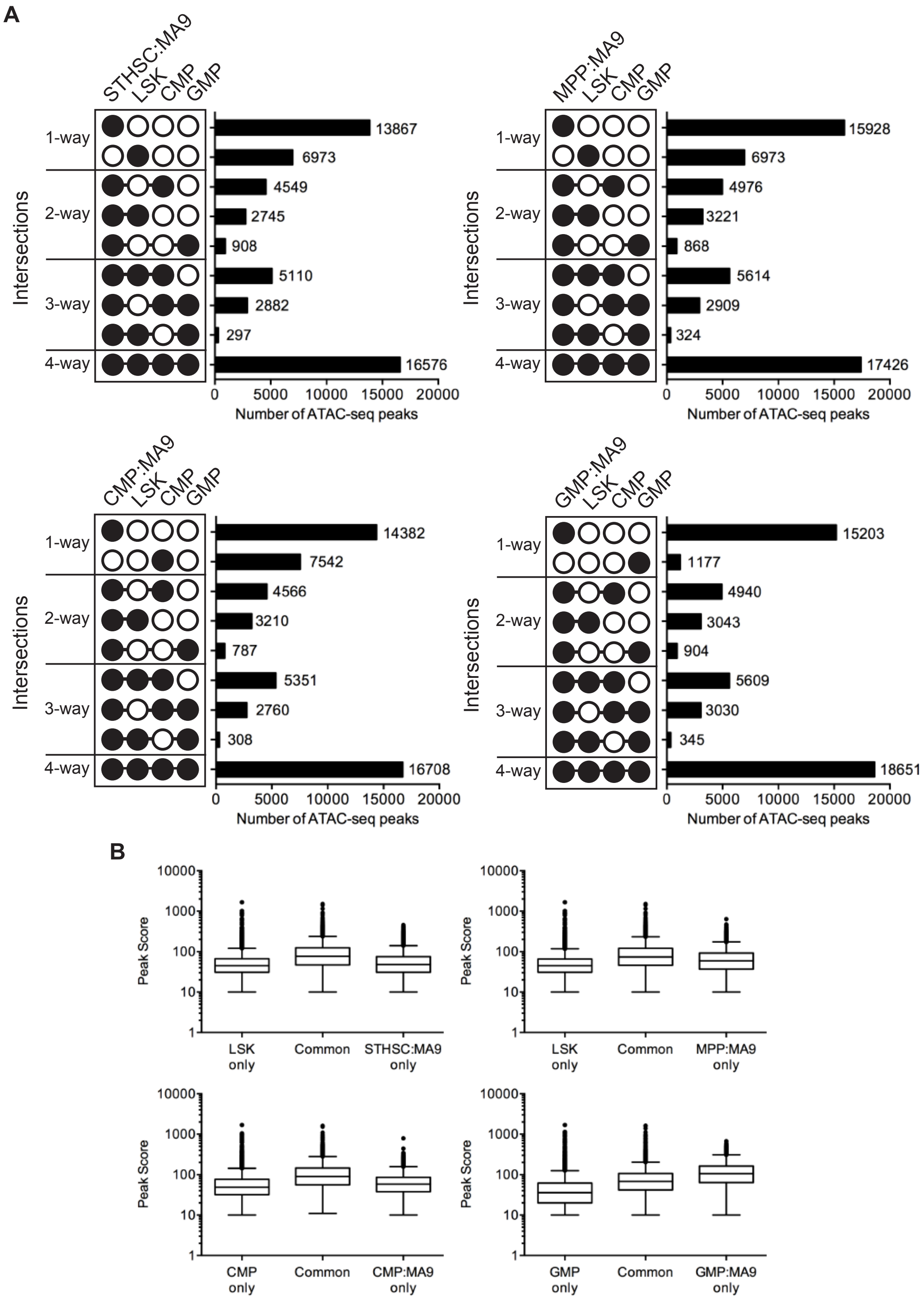

Supplementary Figure 5. Global chromatin remodeling occurs during MLL-AF9 transformation
(A) Number of intersecting ATAC-seq peaks of each leukemia subtype with all normal cell types (LSK; *n* = 3, CMP; *n* = 3, GMP; *n* = 2, STHSC:MA9; *n* = 2, MPP:MA9; *n* = 2, CMP:MA9; *n* = 2, GMP:MA9; *n* = 2). Each sample was obtained from an individual mouse. Data were collected from two biological replicate experiments. LSK; Lin^- $Sca\text{-}1^+$ $c\text{-}Kit^+$, includes ST-HSC and MPP cells. Calculated numbers exclude peaks found in non-selected cell subsets. (B) Tukey-style box-and-whisker plots for unique and overlapping ATAC-seq peak scores (LSK; *n* = 3, CMP; *n* = 3, GMP; *n* = 2, STHSC:MA9; *n* = 2, MPP:MA9; *n* = 2, CMP:MA9; *n* = 2, GMP:MA9; *n* = 2). Each sample was obtained from an individual mouse. Data were collected from two biological replicate experiments. Boxes denote 25th to 75th percentiles.

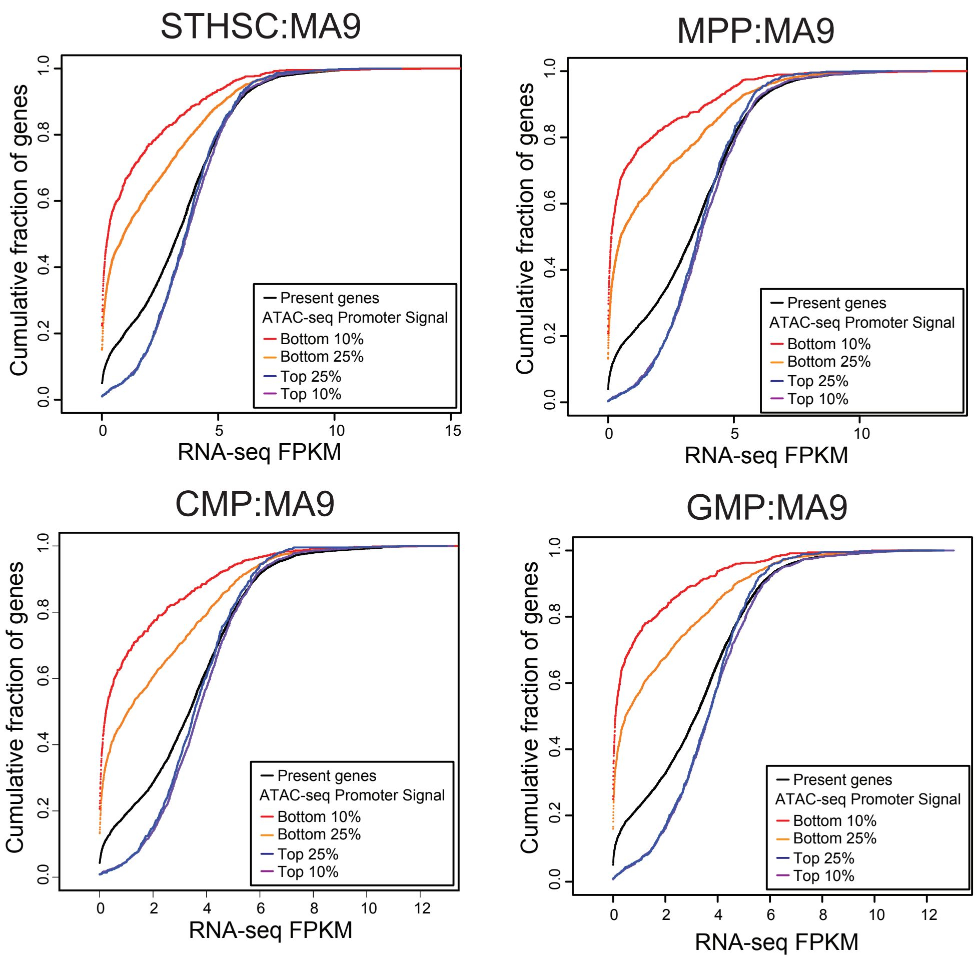

Supplementary Figure 6. Relationship between open chromatin at promoters and local transcript expression RNA-seq FPKM values of genes for which promoter ATAC-seq peak values fall within the bottom 10% of ATAC-seq peaks (red line), the bottom 25% (orange line), all transcripts (black line), top 25% (blue line) and top 10% (purple line). Analysis of single samples are shown. Trends were replicated once with independent biological replicates.

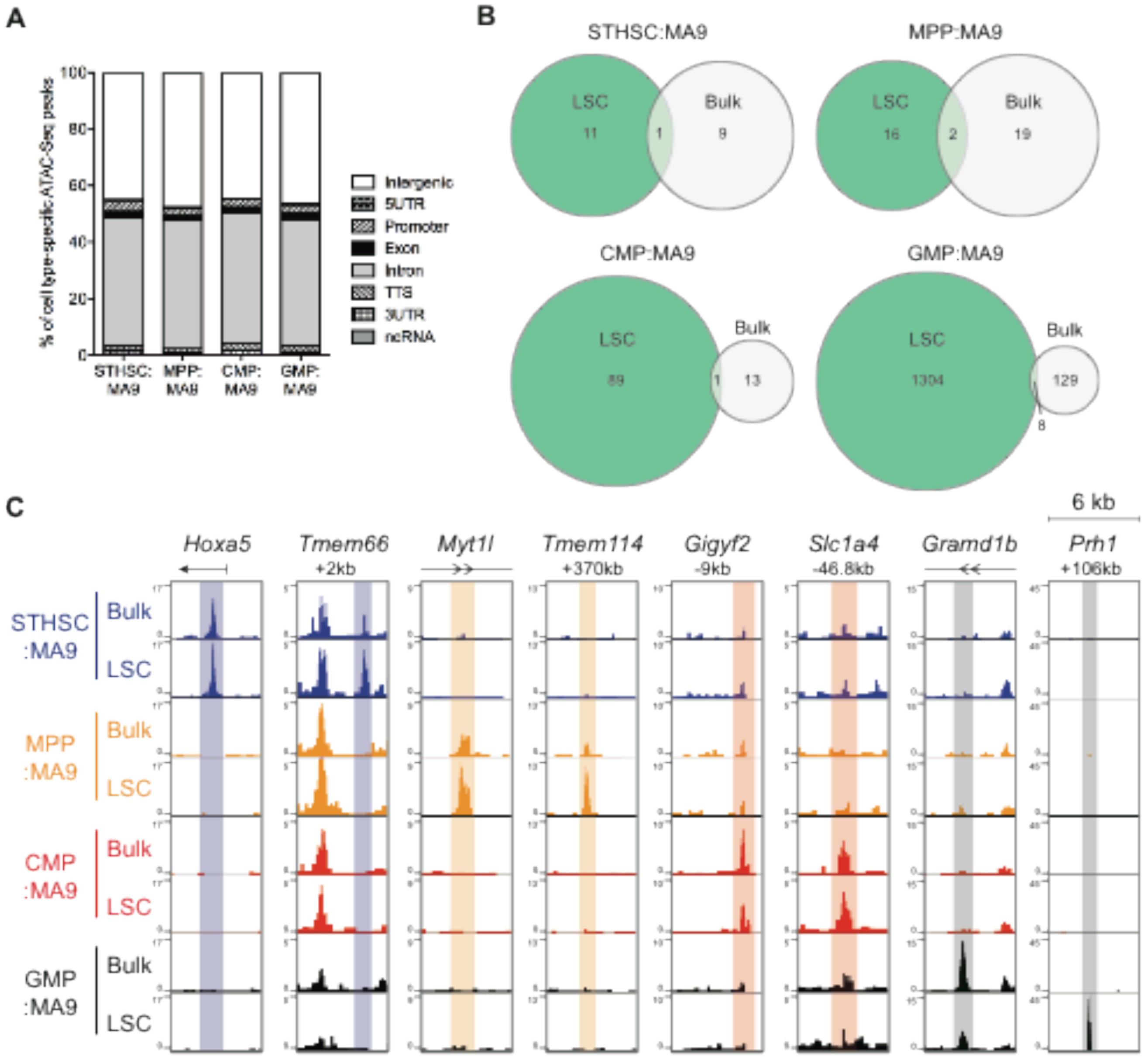

Supplementary Figure 7. Open chromatin regions in bulk AML cells and LSCs (A) Percentage of unique ATAC-seq peaks in bulk leukemias distributed across annotated genomic features (STHSC:MA9; *n* = 2, MPP:MA9; *n* = 2, CMP:MA9; *n* = 2, GMP:MA9; *n* = 2). Each sample was obtained from an individual mouse. Data were collected from two biological replicate experiments. Bars denote mean. (B) Overlap of unique peaks identified in bulk leukemia and LSC samples based on cell-of-origin (STHSC:MA9; *n* = 2, STHSC:MA9 LSC; *n* = 2, MPP:MA9; *n* = 2, MPP:MA9 LSC; *n* = 2, CMP:MA9; *n* = 2, CMP:MA9 LSC; *n* = 2, GMP:MA9; *n* = 2, GMP:MA9 LSC; *n* = 2). Each sample was obtained from an individual mouse. Data were collected from two biological replicate experiments. (C) Normalized ATAC-seq profiles of *in vivo*-derived primary bulk leukemias and LSCs, showing open chromatin regions nearest *Hoxa5*, *Tmem66*, *Myt1l*, *Tmem114*, *Gigyf2*, *Slc1a4, Prh1,* and *Gramd1b* in 6kb regions. Single samples are shown. Trends were replicated once with independent biological replicates.

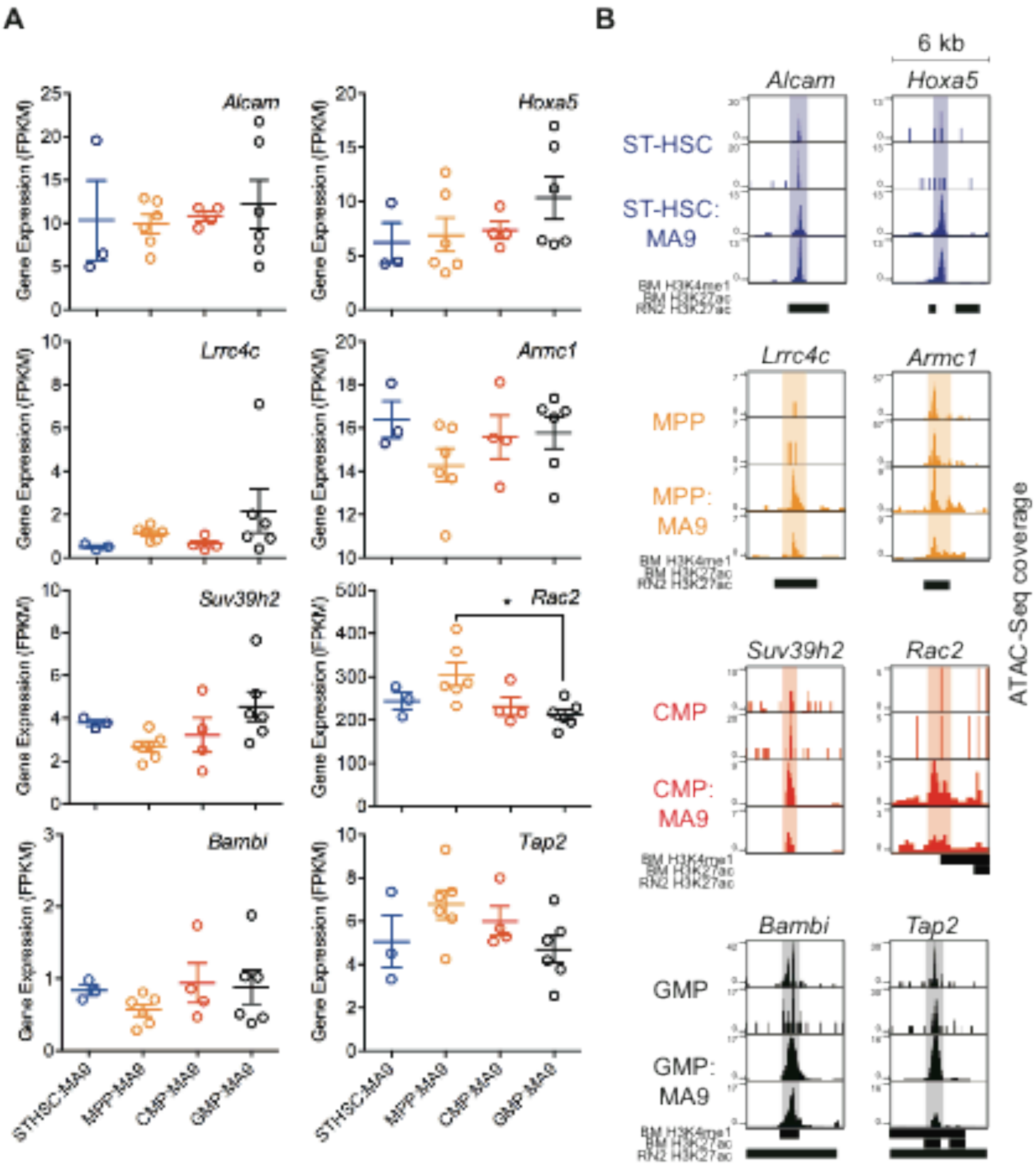

Supplementary Figure 8. Open chromatin regions in bulk AML cells and normal cells-of-origin
(A) Local transcript production (RNA-seq FPKM) nearest to the loci possessing unique open chromatin peaks in bulk leukemias based on cell-of-origin (STHSC; n = 5, MPP; n = 6, CMP; n = 5, GMP; n = 5, STHSC:MA9; n = 3, MPP:MA9; n = 6, CMP:MA9; n = 4, GMP:MA9; n = 6). Each sample was obtained from an individual mouse. Data were collected from two biological replicate experiments. Center bars indicate mean. Error bars indicate s.e.m. Kruskal-Wallis test for *Rac2;* P = 0.0199. Dunn's multiple comparisons test; * $P < 0.05$. (B) Normalized ATAC-seq profiles of *in vivo*-derived primary bulk leukemias and their respective cells-of-origin, showing open chromatin regions nearest *Alcam, Hoxa5, Lrrc4c, Armc1, Suv39h2, Rac2, Bambi* and *Tap2* in 6kb regions. Biological replicates of each are shown. Shown at bottom are H3K4me1 and H3K27ac ChIP-seq peak regions in normal murine bone marrow (BM) and H3K27ac peak regions in an MLL-AF9/Nras^{G12D} murine AML cell line (RN2).

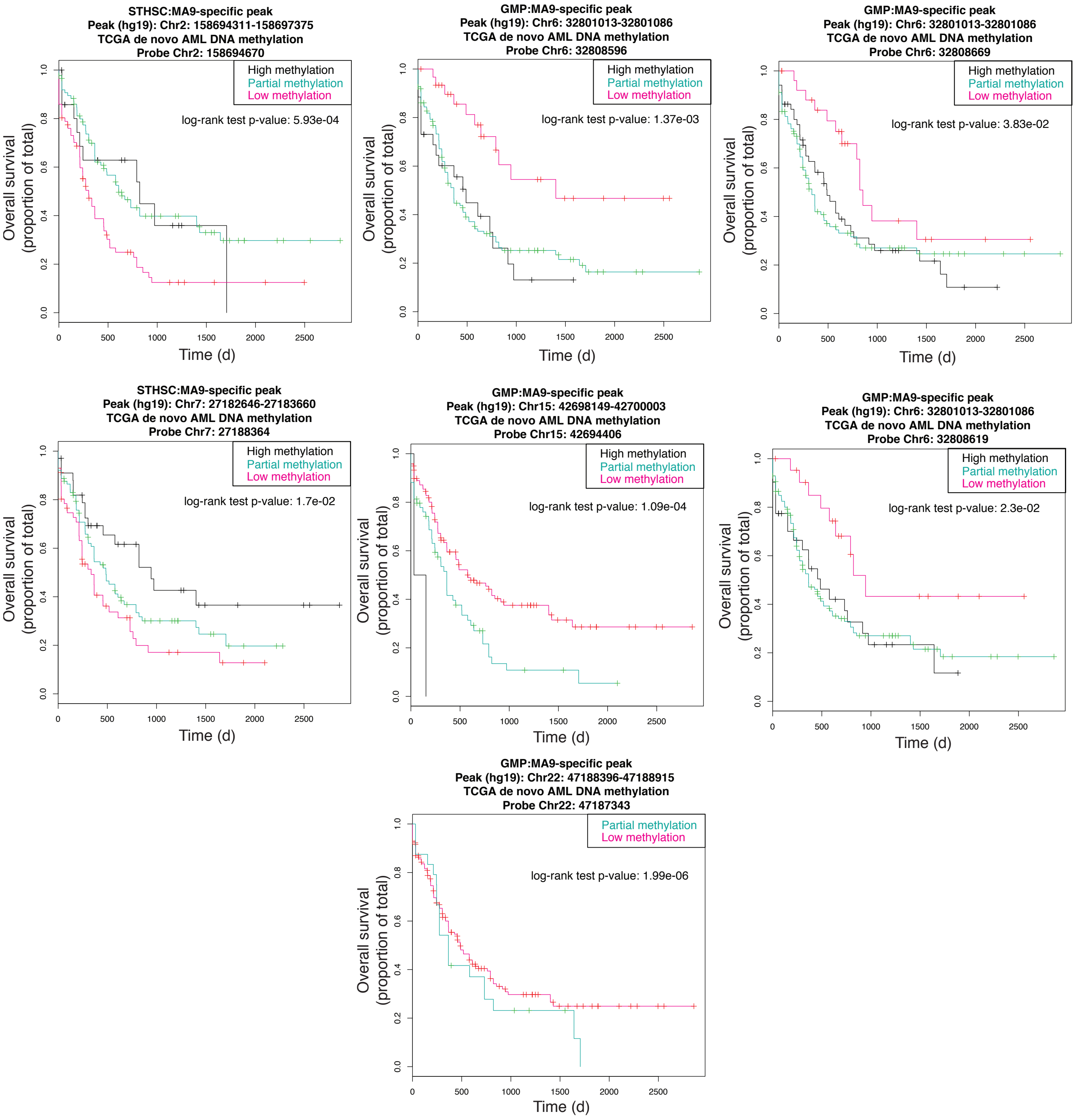

Supplementary Figure 9. DNA methylation loci predictive of outcome in human TCGA AML data and proximal to cell-of-origin-specific open chromatin regions Overall survival of human AML patients based on DNA methylation status of the CpG probe shown (n = 200). Log-rank test P values are denoted on each graph.

Supplementary Table 1. Log-rank (Mantel-Cox) test p values for all pair-wise comparisons of overall survival in mice transplanted with MA9 cell lines as shown in Figure 1C

	100K MA9 (LT)	100K MA9 (ST)	100K MA9 (MPP)	100K MA9 (CMP)	100K MA9 (GMP)
100K MA9 (LT)		0.002	0.002	0.019	0.0116
100K MA9 (ST)	0.002		0.0086	0.1822	0.012
100K MA9 (MPP)	0.002	0.0086		0.4692	0.012
100K MA9 (CMP)	0.019	0.1822	0.4692		0.7419
100K MA9 (GMP)	0.0116	0.012	0.012	0.7419	

Supplementary Table 2. Limiting dilution transplantation data to determine initiating cell frequency

Dose (Cells)	Transplanted	Engrafted	Group
1000	5	1	MA9(LT)
10000	5	3	MA9(LT)
100000	5	5	MA9(LT)
1000	5	0	MA9(ST)
10000	5	3	MA9(ST)
100000	5	5	MA9(ST)
1000	5	1	MA9(MPP)
10000	5	2	MA9(MPP)
100000	5	5	MA9(MPP)
1000	5	0	MA9(CMP)
10000	5	0	MA9(CMP)
100000	5	3	MA9(CMP)
1000	5	0	MA9(GMP)
10000	5	0	MA9(GMP)
100000	5	5	MA9(GMP)

Supplementary Table 3. Pearson's chi-square test p values for all pair-wise comparisons of limiting dilution leukemia initiating cell frequency as shown in Figure 1E

	MA9 (LT)	MA9 (ST)	MA9 (MPP)	MA9 (CMP)	MA9 (GMP)
MA9 (LT)		0.685	0.566	0.000643	0.0501
MA9 (ST)	0.685		0.878	0.00352	0.133
MA9 (MPP)	0.566	0.878		0.00474	0.167
MA9 (CMP)	0.000643	0.00352	0.00474		0.168
MA9 (GMP)	0.0501	0.133	0.167	0.168	

Supplementary Table 4. Log-rank (Mantel-Cox) test p values for all pair-wise comparisons of overall survival in mice transplanted with MA9-transduced primary cells as shown in Figure 2B

	STHSC:MA9	MPP:MA9	CMP:MA9	GMP:MA9
STHSC:MA9		0.2319	0.0358	<0.0001
MPP:MA9	0.2319		0.1634	<0.0001
CMP:MA9	0.0358	0.1634		0.0086
GMP:MA9	<0.0001	<0.0001	0.0086	

Supplementary Table 5. Somatic mutations identified in coding regions of bulk tumor samples

**Leukemia Cell Type** (# samples)	**STHSC:MA9** (3)	**MPP:MA9** (6)	**CMP:MA9** (4)	**GMP:MA9** (6)
1700128F08Rik	-	2[a]	-	-
C130026I21Rik	-	1[b]	-	-
Csde1	-	1[c]	-	-
Ctsa1	-	1[d]	-	-
Eef1b2	-	1[e]	-	-
Fam111a	-	1[f]	-	-
Fibp	-	1[g]	-	-
Gm21092	-	1[h]	-	-
Gm340	-	1[i]	-	-
Hist1h2br	1[j]		-	-
mt-Co1	-	1[k]	-	-
Odc1	-	1[l]	-	-
Prdx5	-	1[m]	-	-
Prex1	-	1[n]	-	-
Samhd1	-	1[o]	-	-
Sec61b	-	1[p]	-	-
Sephs1	-	1[q]	-	-
Setdb1	-	1[r]	-	-
Sf3b3	-	1[s]	-	-

[a]p.TyrTyr141* (stop), p.TyrTyrLeu141* (stop); [b]p.Arg56* (LOF, NMD); [c]p.Glu740* (LOF, NMD); [d]p.ThrSer2* (LOF, NMD); [e]p.Glu111* (LOF, NMD); [f]transcript variant; [g]p.Glu217* (LOF, NMD); [h]p.Lys208* (stop); [i]p.Glu791* (stop); [j]pMetPro1? (LOF); [k]p.Ter6Serext*? (stop lost); [l]p.Trp356* (LOF, NMD); [m]p.Met1? (start lost); [n]p.Gln797* (LOF, NMD); [o]p.Ser28* (LOF, NMD); [p]p.CysGlnLeu58* (LOF, NMD); [q]p.Gly156* (LOF, NMD); [r]p.Gln445* (LOF, NMD); [s]p.Gln651* (LOF, NMD)
